# Molecular Characterization of Arbuscular Mycorrhizal Fungi in an Agroforestry System Reveals the Predominance of *Funneliformis* spp. Associated with *Colocasia esculenta* and *Pterocarpus officinalis* Adult Trees and Seedlings

**DOI:** 10.3389/fmicb.2017.01426

**Published:** 2017-07-28

**Authors:** Alexandre Geoffroy, Hervé Sanguin, Antoine Galiana, Amadou Bâ

**Affiliations:** ^1^Laboratoire de Biologie et Physiologie Végétales, L’Unité de Formation des Sciences Exactes et Naturelles, Unité Mixte de Recherche LSTM, Université des Antilles Pointe-à-Pitre, France; ^2^Unité Mixte de Recherche LTSM, Centre de Coopération Internationale en Recherche Agronomique Pour le Développement Montpellier, France

**Keywords:** arbuscular mycorrhizal community, *Colocasia esculenta*, *Funneliformis*, Guadeloupe, *Pterocarpus officinalis*, pyrosequencing, tropical agroforestry

## Abstract

*Pterocarpus officinalis* (Jacq.) is a leguminous forestry tree species endemic to Caribbean swamp forests. In Guadeloupe, smallholder farmers traditionally cultivate flooded taro (*Colocasia esculenta*) cultures under the canopy of *P. officinalis* stands. The role of arbuscular mycorrhizal (AM) fungi in the sustainability of this traditional agroforestry system has been suggested but the composition and distribution of AM fungi colonizing the leguminous tree and/or taro are poorly characterized. An in-depth characterization of root-associated AM fungal communities from *P. officinalis* adult trees and seedlings and taro cultures, sampled in two localities of Guadeloupe, was performed by pyrosequencing (GS FLX+) of partial 18S rRNA gene. The AM fungal community was composed of 215 operational taxonomic units (OTUs), belonging to eight fungal families dominated by Glomeraceae, Acaulosporaceae, and Gigasporaceae. Results revealed a low AM fungal community membership between *P. officinalis* and *C. esculenta*. However, certain AM fungal community taxa (10% of total community) overlapped between *P. officinalis* and *C. esculenta*, notably predominant *Funneliformis* OTUs. These findings provide new perspectives in deciphering the significance of *Funneliformis* in nutrient exchange between *P. officinalis* and *C. esculenta* by forming a potential mycorrhizal network.

## Introduction

*Pterocarpus officinalis* L. is one of the dominant wetland tree species of the seasonally flooded swamp forests in the Caribbean and the Guiana regions (Eusse and Aide, 1999; Bâ and Rivera-Ocasio, 2015). It covers large areas of the coastal floodplain as individual trees and small patches adjacent to mangroves, and along rivers and in mountains (Eusse and Aide, 1999). In the Caribbean, this unique *P. officinalis* swamp forest provides a habitat for many species of plants and animals and reduces soil erosion along the margins and riverbanks in coastal and mountain areas (Saur and Imbert, 2003; Bâ and Rivera-Ocasio, 2015). Despite its ecological interest, most of the populations of *P. officinalis* in the Caribbean islands are restricted to a small area due to wetland drainage and urban development (Bâ and Rivera-Ocasio, 2015). Furthermore, the low genetic diversity found within and between populations of *P. officinalis* is exacerbated by a strong inbreeding depression (Muller et al., 2009). As a consequence, management and conservation measures must be implemented to preserve the remaining *P. officinalis* populations.

In some Caribbean islands, a dominant management strategy for *P. officinalis* conservation is to plant agricultural crops under these stands. The Guadeloupean smallholder farmers notably conduct taro (*Colocasia esculenta* L. Schott) monocultures under the *P. officinalis* stands in freshwater flooding swamp forests because of higher crop yields compared to other agricultural practices (Saur and Imbert, 2003). No fertilizer or pesticide are used, and the *P. officinalis* trees are not impacted (no cutting), preserving the tree genetic diversity, and the ecosystem processes (e.g., nutrient cycling, local biodiversity) (Rivera-Ocasio et al., 2006; Bâ and Rivera-Ocasio, 2015). The ecological mechanisms sustaining the functioning of this traditional agroforestry system remains poorly investigated. Indeed heavy leaching of soils brought by seasonal flooding contributes to the shortage of available P and N and should be detrimental for taro monocultures (Bâ and Rivera-Ocasio, 2015). *Pterocarpus* stands might be beneficial to understory taro cultures by (i) maintaining humidity and temperature at certain levels to prevent water stress (Sanou et al., 2012), and (ii) improving the N input on soil and non-legume plants through biological nitrogen fixation process (Koponen et al., 2003; Saur and Imbert, 2003). The transfer of N from legume to the non-legume can occurred through root exudation, root and nodule decomposition and mineralization, as well as mediated by plant-associated arbuscular mycorrhizal (AM) fungi (Kaiser et al., 2015). Both *P. officinalis* and *C. esculenta* establish a symbiosis with AM fungi (Saint-Etienne et al., 2006; Wang and Qiu, 2006; Fougnies et al., 2007), notably improving plant phosphorus (P) nutrition (Smith and Read, 2008), but also potentially plant N nutrition (Hodge et al., 2001; Walder et al., 2012). In addition, *P. officinalis* requires P from AM fungi not only for their nutrition but also for efficient nodule formation and nitrogen fixation (Fougnies et al., 2007; Le Roux et al., 2014). Whereas, the diversity of nitrogen-fixing bacteria associated with *P. officinalis* in Caribbean swamp forests has been described (Le Roux et al., 2014), AM fungal diversity has been poorly investigated.

Arbuscular mycorrhizal fungi are the most common and widespread symbiosis involving 86% of land plants including many important crops (Davison et al., 2012). The extraradical phase of AM fungi acts as an extension of the root system for the uptake of nutrients in exchange for plant-synthesized carbon. AM fungi are assumed to exhibit non-specific symbiosis but a given AM taxa could have different effects depending on plant species (Thioye et al., 2017). The relatively low host specificity of AM fungi, increases the possibility that extraradical fungal hyphae links multiple plant species to form common mycorrhizal networks (CMNs) in a plant community (Cheng and Baumgartner, 2004; Ingleby et al., 2007). Mycorrhizal networks are known to drive nutrient transfers (mainly C and N) between adult trees and seedlings for one plant species, and among different plants species (e.g., legume and non-legume) (Cheng and Baumgartner, 2004; He et al., 2004; Selosse et al., 2006; Wahbi et al., 2016). However, mycorrhizal networks have been mainly assessed at the fungal strain level in controlled conditions (Walder et al., 2012) and rarely at the community level (Montesinos-Navarro et al., 2012). Thanks to the development of high-throughput sequencing approaches such as pyrosequencing, the complexity of AM fungal community among plant roots can be deeply assessed (Hart et al., 2015), and a wide range of ecological studies based on the diversity of AM fungal taxonomic markers such as the SSU rRNA gene has been performed (Öpik et al., 2006, 2014).

The current study aims the molecular characterization of AM fungal community composition and distribution between *P. officinalis* and *C. esculenta* crops in a traditional Guadeloupean agroforestry system (swamp forests) in order to evaluate if the sustainability of the system might be explained by a high similarity or a high dissimilarity of AM fungal community between *Pterocarpus* and taro. In addition, the comparison of AM fungal community of *P. officinalis* adult trees and seedlings with the ones of taro were investigated because of the potential importance of seedlings in the sustainability of the agroecosystems since they are conserved inside the taro cultures. Consequently, two main questions were assessed, (i) What are the predominant mycorrhizal taxa in the traditional agroforestry system, and (ii) What is the degree of similarity of AM fungal members among *P. officinalis* adult trees, seedlings and taro?

## Materials and Methods

### Study Sites and Sampling

We conducted the sampling during 2012 in two representative *Pterocarpus* swamp forests located in the Grande-Terre island of Guadeloupe: Grande Ravine (GR) (16°13′N, 61°28′W) and Belle Plaine (BP) (16°17′N, 61°31′W) (**Supplementary Figure S1**). The *P. officinalis* stands, which comprise 45 and 52 adult trees for GR and BP sites, respectively, are fairly forested and contain dense populations of understory regenerating seedlings. The GR forest site (approximately 0.3 ha) is located along the GR river and taro plants (*C. esculenta*) were cultivated by smallholders farmers under adult trees and between naturally regenerating seedlings. Some individuals of understory plant species such as *Ficus* sp.1, *Commelina* sp.1, and *Mimosa pudica* were naturally associated with *P. officinalis*. The BP forest site (approximately 0.4-ha), is located around the bay of the Grand Cul-de-sac Marin, in the near mangrove area and taro plants were also cultivated between *Pterocarpus* trees and regenerating seedlings. Understory species like *Musa* sp. and *Ficus* sp. are represented by a few individuals and are widely spaced from one another. In each forest site, soil cores (200 g of fresh soil) were randomly collected near three adult trees (more than 25 m high), three seedlings (1 < height < 2 m) and three taro plants. Overall, 18 soil cores were stored at 4°C before being processed. Roots were separated from soil, gently washed with tap water and dried with Silica-gel until molecular analyses. Soil physico-chemical parameters were measured at the Celesta-lab (Mauguio, France) (Supplementary Table S1).

### Molecular Analyses

For each root sample, the three replicates were pooled and subjected to liquid nitrogen grinding for homogenization. Total DNA was extracted from a sub-sample (100 mg of dried root) using a FastPrep-24 homogenizer (MP Biomedicals Europe, Illkirch, France) and the FastDNA^®^ SPIN kit (MP Biomedicals Europe) according to manufacturer’s instructions. The quality of DNA extracts was improved by adding 20–30 mg Polyvinylpolypyrrolidon (PVPP) during the first step of DNA extraction. Two DNA extractions were done per root sample.

*Glomeromycota* (AM fungi) sequences were amplified using the nuclear 18S rRNA gene primers NS31 [5′- (10-bp MID) TTGGAGGGCAAGTCTGGTGCC -3′] (Simon et al., 1992) and AML2 [5′- (10-bp MID) GAACCCAAACACTTTGGTTTCC -3′] (Lee et al., 2008). MIDs (multiplex identifier) were designed by Eurofins Genomics (Eurofins Genomics GmbH^1^). PCR conditions were performed according to Sanguin et al. (2016). Two PCR products per root sample were pooled before purification using illustra GFX PCR DNA and Gel Band Purification Kit (GE Healthcare Life Sciences, Velizy-Villacoublay, France) following manufacturer’s guidelines. Overall 18 PCR products were subjected to bi-directional 454-sequencing (1/4th plate Roche GS FLX+ run using the GS FLX Titanium sequencing kit XL+) by Eurofins Genomics.

### Data Processing and Taxonomic Assignment of AM Fungal Sequences

Four hundred and fifty-four-sequencing data were analyzed using Mothur software according the standard operating procedure^2^ proposed (Schloss et al., 2011), except for the quality cutoffs, for which it has been set up at Q30 (*trim.seqs* command). All sequence reads were then depleted of barcodes and primers (final length 230 bp), and sequences < 100 bp or with ambiguous base calls or with homopolymer runs exceeding 8 bp were also removed. A pre-clustering step (Huse et al., 2010) was also performed to remove sequences still likely due to pyrosequencing errors. Chimeric sequences were checked by using UCHIME (Edgar et al., 2011) and removed. Finally, sequences were identified using the Glomeromycota-based alignment database (Krüger et al., 2012) and sequence similarity ≥60% at the family level.

Clustering of sequences in operational taxonomic unit (OTUs) was performed using *dist.seqs* and cluster commands in Mothur. Then, the number of sequences from each sample was normalized with *sub.sample* command. This sub-sampling step allows reducing the number of spurious OTUs and is crucial to obtain robust estimation of alpha and beta diversity (Gihring et al., 2012). Finally, OTUs were defined at 97% similarity level for taxonomic affiliation.

### Statistical Analyses

Diversity (Shannon, inverse Simpson [1/D]), richness (number of OTUs, Chao1) and evenness (Pielou) indexes were estimated using R version 3.3.2 (R Core Team, 2016) and the R package vegan (Oksanen et al., 2016). The sequencing effort was evaluated using the coverage calculator and Boneh estimator (Boneh et al., 1998) implemented in Mothur. We assessed the effects of forest site, plant species, *Pterocarpus* age categories and their interactions on the AM fungal community composition by non-parametric permutational multivariate analysis of variance (PERMANOVA) (Anderson, 2001) implemented in the *perm.anova*() function from the R package RVAideMemoire (Hervé, 2016). The differences in AM fungal community structure among forest sites and plant species were assessed using PERMANOVA in *adonis*() function (McArdle and Anderson, 2001), both from the R package vegan. The AM fungal community structure was based on the Bray–Curtis dissimilarity index as defined in *vegdist*() function from the R package vegan. Multivariate dispersion was estimated using the *betadisper*() and *permutest*() functions (999 permutations; alpha = 0.05) from the R package vegan because it can affect PERMANOVA results. Differences in the relative abundances of AM fungal OTUs among forest sites or plant species were estimated using Kruskal–Wallis’ test implemented in *kruskal.test*() function from the R package stats. The frequency of AM fungal OTUs was determined using the *strassoc*() function from the R package indicspecies (De Cáceres and Legendre, 2009).

The determination of AM fungal OTUs preferentially associated with a given forest site was performed using the corrected Pearson’s phi coefficient of association (“r.g”) implemented in the *multipatt*() function (De Cáceres et al., 2010) from the R package indicspecies. AM fungal OTUs preferentially associated with a given plant species was assessed using the corrected indicator value index (“IndVal.g”species) from the R package indicspecies. A procedure based on determination of species (i.e., OTU) and group (i.e., plant type) combinations was applied using successively *combinespecies*() and *multipatt*() functions. This procedure was demonstrated to bear more ecological informations and to determine more robust predictive indicator value than by considering species or group independently (De Cáceres et al., 2012). Two different probabilities were calculated, i.e., A (specificity), representing the probability of a sample to be defined by a group (i.e., plant type), given that the species or the species combinations have been detected, and B (sensitivity) representing the probability of finding the species or the species combinations in different samples characterized by a given group (i.e., plant type). Only AM fungal OTUs present in two samples among three groups defined (i.e., plant type) were subjected to analysis. We considered as valid indicators the OTUs showing both A (specificity) and B (sensitivity) superior to 0.8 and 0.6, respectively, as recommended in Suz et al. (2014).

The AM fungal community membership among *C. esculenta* and *P. officinalis* adult trees and seedlings was assessed using venn diagram analysis with the R package VennDiagram (Chen, 2016), and the relative abundance of AM fungal taxa shared among plants was characterized using bipartite network analysis with the *plotweb*() function from the R package bipartite (Dormann et al., 2008).

## Results

### AM Fungal Community Composition among Forest Sites

The global 454-pyrosequencing data were composed of 210,676 reads, and 155,544 reads (74%) passed the quality control steps. The average read length was 230 bp. After trimming, pre-clustering and chimera detection steps, 70,949 sequences were classified using a Glomeromycota-based alignment database. A total of 31,803 non-Glomeromycota sequences were removed from the dataset (70,949), as well as singletons (1515 sequences) that are mostly considered as artifacts and can lead to overestimations of AM fungal diversity. The AM fungal sequences (37,631 reads) were assigned to a total of 215 OTUs based on a sequence similarity threshold ≥ 97%. The sequence number between samples was rarefied to 440 sequences per sample (threshold based on the sample with the lower number of sequences) to improve statistical robustness. A high diversity coverage (94–98%) was reached for all samples, with less than eight potential OTUs that were not retrieved (Boneh estimation, Supplementary Table S2). The Boneh estimation showed that the sequencing depth was sufficient to estimate and compare the microbial diversity of the samples (Supplementary Table S2).

The taxonomy assignment of OTUs (Supplementary Table S3) revealed a main affiliation to Glomeraceae (83.5% of sequences), but also to Acaulosporaceae (9.7%), Gigasporaceae (5.3%), Archaesporaceae (0.9%), Geosiphonaceae (0.3%), Diversisporaceae (0.1%), Paraglomeraceae (0.05%), and Pacisporaceae (0.03%). In Glomeraceae, *Funneliformis* represents the most abundant genus (62% of sequences), followed by fungi of uncertain position in *Glomus* sensus lato (20%), *Rhizophagus* (2%), and *Sclerocystis*/*Glomus* (<1%) (Supplementary Table S3). The low evenness estimated for all AM fungal communities (<0.3) revealed the strong predominance of few OTUs and numerous rare OTUs (Supplementary Table S2). Three OTUs, i.e., OTU1 and OTU2, belonging to Glomeraceae and OTU3 to Acaulosporaceae families, represented more than 80% of sequences (Supplementary Table S3). The most predominant OTU (OTU1, 58% of sequences) was affiliated to *Funneliformis*.

**FIGURE 1:**
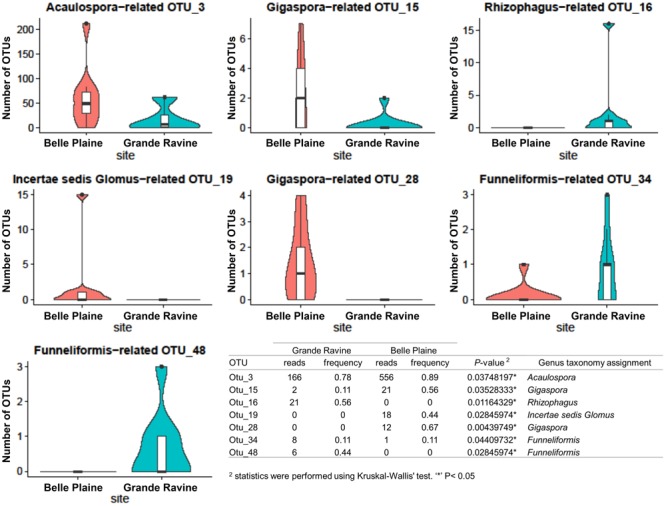
Abundance of arbuscular mycorrhizal (AM) fungal operational taxonomic units (OTUs) between Grande Ravine and Belle Plaine forest sites. Only OTUs with significant (*P* < 0.05) differences in their relative abundance between the both forest sites are shown. Table represented comparison of AM fungal OTU abundances between localities (Belle Plaine and Grande Ravine) with statistics were performed using Kruskal–Wallis’ test. ^∗^*P* < 0.05.

Richness, diversity and evenness were calculated for the different AM fungal communities. No significant difference was observed at both forest sites (Supplementary Table S2). AM fungal community structure analysis, based on Bray–Curtis index, also showed no significant difference between both forest sites (**Table 1**). However, four OTUs affiliated to *Acaulospora, Gigaspora*, and *Incertae sedis Glomus* showed significantly higher abundance in BP, and three OTUs affiliated to *Rhizophagus* and *Funneliformis* in GR (**Figure 1**, for all comparisons see Supplementary Table S4). Among the 215 AM fungal OTUs, only four were determined as preferentially associated at a given forest site (Supplementary Table S5), among which two of them, i.e., *Gigaspora* (OTU28) and *Rhizophagus* (OTU16) were exclusively found in BP and GR sites, respectively.

**Table 1:** Impact of locality and plant type on arbuscular mycorrhizal (AM) fungal community structures in agroforestry systems.

Locality	Factors	Df	SS	MS	F. Model	*R*^2^/N perm	*P*-value^1^
All	Locality	1	0.11671	0.11671	1.298	0.05730	0.242^ns^
	Plant	2	0.26401	0.132006	1.4610	0.12962	0.173^ns^
	Locality × Plant	2	0.57188	0.285939	3.1647	0.28077	**0.011^∗^**
	Residuals	12	1.08422	0.090352		0.53231	
	Total	17	2.03682			1	
Belle Plaine	Plant	2	0.38777	0.19389	1.4766	0.32984	0.079^ns^
	Residuals	6	0.78786	0.13131		0.67016	
	Total	8	1.17563			1.00000	
Grande Ravine	Plant	2	0.44812	0.224058	4.5362	0.60192	**0.036^∗^**
	Residuals	6	0.29636	0.049393		0.39808	
	Total	8	0.74448			1.00000	

*^1^The significance of multivariate analysis of variance was assessed using PERMANOVA with *adonis*() function (iterations = 999 permutations). ^∗^*P* < 0.05, ^ns^*P* > 0.05. Multivariate dispersion was tested using the *betadisper*() and *permutest*() functions (iterations = 999 permutations; alpha = 0.05) revealing a significant homogeneity of group dispersions.*

### AM Fungal Community Composition among Plant Types

Arbuscular mycorrhizal fungal community richness (for the two indices Chao1 and OTUs number) was significantly different between taro and *Pterocarpus* (*P* = 0.033; Supplementary Table S2) whereas only a locality-dependent plant type effect was observed on AM fungal community structures (*P* = 0.011; **Table 1**). The analysis of AM fungal community structure for each forest site confirmed the plant type effect for the GR forest site (*P* = 0.036) (**Table 1**). The NMDS analysis (**Supplementary Figure S2**), taking into account all OTUs, highlighted the dissimilarity among AM fungal communities of *Pterocarpus* adult trees compared to both taro and *Pterocarpus* seedlings in the GR site. Out of a total of 7750 and 9730 AM fungal OTUs and OTU pair combinations for the BP and GR sites, 8 and 27 AM fungal indicators were determined, respectively (**Table 2** and Supplementary Table S6), confirming the significant and preferential association of *Gigaspora* (OTU22 and OTU33) with taro. In the GR forest site, six AM fungal OTUs (OTU1, OTU2, OTU4, OTU5, OTU6, and OTU48) belonging to the Glomeraceae were significantly associated with both taro and *Pterocarpus* seedlings, and two AM fungal OTUs (OTU18 and OTU3) with *Pterocarpus* adult trees.

**Table 2:** Single of AM fungal operational taxonomic units (OTUs) associated with a plant type in *Pterocarpus*-taro in Guadeloupean agroforestry systems.

Locality	Plant	Indicator taxa	Frequency^1^	*A*^2^	*B*	IndVal.g	*P*-value^3^
		Taxonomy (OTU)	(P.a/P.s/T)	(specificity)	(sensibility)		
Belle Plaine	T.	*Gigaspora (33)*	0.0/0.0/1.0	1.00	1.00	1.00	0.027^∗^
Grande Ravine	T.	*Gigaspora (22)*	0.0/0.0/1.0	1.00	1.00	1.00	0.035^∗^
	P.a	*Geosiphon (18)*	0.0/0.0/1.0	1.00	1.00	1.00	0.032^∗^
		*Archaeospora (10)*	1.0/0.0/0.3	0.96	1.00	0.98	0.032^∗^
	T. +P.s	*Incertae sedis Glomus (4)*	0.0/1.0/1.0	1.00	1.00	1.00	0.032^∗^
		*Rhizophagus (23)*	0.0/1.0/1.0	1.00	1.00	1.00	0.032^∗^

*^1^Frequency indicates the presence of an OTU in samples of a given plant type. P.a, *Pterocarpus* adult tree; P.s, *Pterocarpus* seedling; T., taro. ^2^The corrected Indval coefficient of association (“IndVal.g”) was used as model to determine indicator OTUs. ^3∗^*P* < 0.05, ^ns^*P* > 0.05.*

**FIGURE 2:**
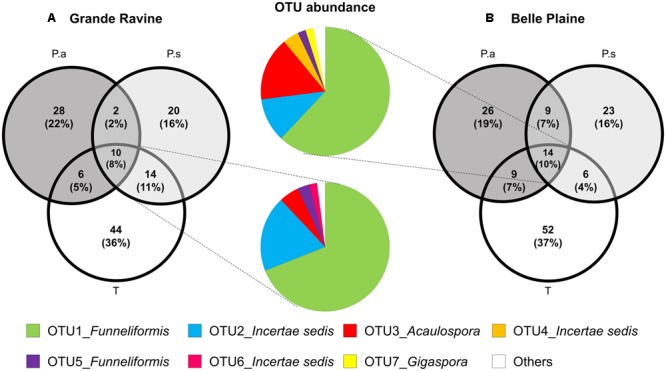
Distribution of AM fungal OTUs shared among taro (T), *Pterocarpus* adult trees (P.a) and seedlings (P.s) in **(A)** Grande Ravine and **(B)** Belle Plaine forest sites. All sequences were clustered in OTUs (97% similarity). Data were rarefied to 440 sequences per sample. Venn diagrams represent the percentage of AM fungal OTUs specific to a given plant or overlapping among plants. Color pie charts represents the abundance of OTUs shared among the three plants for both forest sites. The category “Others” combines OTUs representing less than 1% (8 OTUs for BP and 5 for GR).

Venn diagram analysis of OTU-based AM fungal community revealed a low membership characterized by a high number of OTUs specific to one plant and few overlapping OTUs among taro and the two age categories of *Pterocarpus*, i.e., 10 and 8% of common OTUs in BP and GR sites, respectively (**Figure 2A**). Taro showed the highest number of specific OTUs (>35%) compared to *Pterocarpus* (<25%) in both forest sites. Bipartite network analysis showed that the overlapping AM fungi between plants species was mainly composed of *Funneliformis* OTUs, with 53 and 61% of sequences in BP and GR forest sites, respectively (**Figure 2B**). Rare OTUs mainly constituted the plant-specific OTUs, which fit with the low number of indicator taxa associated with the different types of plants.

## Discussion

Arbuscular mycorrhizal fungi might play a major role in the functioning of the traditional Guadeloupean agroforestry system associating *P. officinalis* trees, their naturally regenerating seedlings, and an understory taro monoculture, and the degree AM fungal community similarity among plants has been hypothesized as one of the main factors. Significant differences in AM fungal community richness and structure were observed among the different plant types, but not in terms of diversity. Some AM fungal OTUs were preferentially associated with a given plant type, but a highly predominant AM fungal OTU affiliated to *Funneliformis* was detected among all plants.

### Characteristics of AM Fungal Community

A relatively high AM fungal richness (>120 OTUs) was observed compared to other AM fungal surveys using high-throughput methods from a wide range of in tropical forest ecosystems (22–207 OTUs; De Beenhouwer et al., 2015; Holste et al., 2016; Rodríguez-Echeverría et al., 2016). As shown by Bainard et al. (2011), tree-based cropping systems, combining different tree species (white ash, hybrid poplar and Norway spruce) with annual crops (corn, soybean, and winter wheat), can present a highly diverse AM fungal community. However, the robustness of comparisons between different tropical agro-ecosystems remains questionable due to the scarcity of studies in tropical regions. Furthermore, OTU-based fungal richness is highly dependent on the bioinformatics treatment applied (mainly sequence quality filtering and clustering methods) (Bálint et al., 2016); the biological material analyzed (roots, spores, and extraradical mycelium) (Varela-Cervero et al., 2015) or the methodology used (spore identification, PCR-cloning, pyrosequencing) (Davison et al., 2015). The predominance of Glomeraceae, which is the most widespread family in natural and managed ecosystems (Oehl et al., 2010; Brearley et al., 2016) and Acaulosporaceae was in agreement with several surveys carried out in tropical environments (Leal et al., 2013; De Beenhouwer et al., 2015; Holste et al., 2016). Only 4% of all detected AM fungal OTUs were poorly affiliated to a reference taxa in databases, which contrasted with previous data in dry afromontane forests (Wubet et al., 2003) and dry tropical regions (Rodríguez-Echeverría et al., 2016), where up to 18 and 15% of OTUs were considered as new taxa, respectively.

Characteristics of AM fungal community (diversity, richness, and structure) were relatively comparable in this cropping system between the two forest sites (separated of 15 km). However, some AM fungal were associated to a given forest site, notably for BP site. The soil characteristics and soil hypoxia are known as major drivers of AM fungal community (Helgason and Fitter, 2009; Oehl et al., 2010; Alguacil et al., 2016) and differences observed between the both forest sites in soil nutrient contents (N, Ca, and Na; Supplementary Table S1) and flooding duration might have favored specific taxa. The low number of indicator species observed in our work was consistent with the study by Moora et al. (2014), which showed that forest plantations or cultivated lands have very few AM indicator species compared to primary forests or permanent grasslands.

### Degree of AM Fungal Community Similarity between Tree and Culture

The analysis of AM fungal community composition and structure demonstrated a site-dependent host plant effect due to low abundant OTUs notably belonging to *Gigaspora* for taro, *Geosiphon* and *Archaeospora* for *Pterocarpus* adult trees. In addition, the significant differences observed between *Pterocarpus* adult trees and seedlings confirmed the modification of AM fungal communities according to the plant age (Wubet et al., 2009). Our data corroborate previous molecular studies conducted in tropical forests where divergent AM fungal communities of co-occurring plant species were reported (Uhlmann et al., 2004; Wubet et al., 2009; Mangan et al., 2010). However, three highly abundant AM fungal taxa (80% of sequences) were associated to the three plant types, notably *Funneliformis* (OTU1) that is considered as a generalist AM fungus (Öpik et al., 2006). *Funneliformis* was shown to form CMNs for transport of N, particularly in tropical environments where N is poorly available (Walder et al., 2012; Munroe and Isaac, 2014). CMNs might play a major role in the sustainability of *Pterocarpus*-taro agroforestry systems and high crop yields. First, the CMNs maintained by *Pterocarpus* could provide an AM fungal mycelium reservoir enabling a faster colonization of short-lived crops under swamp forests (Kuyper et al., 2004) compared to an AM spore reservoir (Brundrett and Abbott, 1994). Secondly, the CMNs could be involved in the N transfers from *Pterocarpus* trees or seedlings to taro in N-deficient soils of swamp forests, as observed between other legume and non-legume associations (Thilakarathna et al., 2016).

**FIGURE 3:**
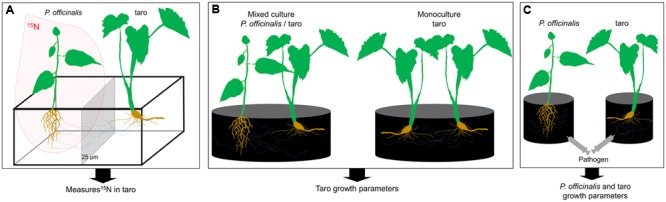
Conceptual experimental design to evaluate role of *Funneliformis* in the nitrogen transfer from *Pterocarpus* to taro through CMNs **(A)**, in the potential benefit of *Pterocarpus-taro* associations **(B)**, and in plant pathogen defense **(C)**. For the three experimental design, all compartments are filled with sterile growth substrate and amended with nutritive solution [without N for **(A)**]. Inoculated (*Funneliformis* strain) and non-inoculated (control), are compared. **(A)** Microcosms, consisting of two plant individuals, set up in compartmented containers subdivided by nylon mesh screens (25 μm). This type of screen is pervious for fungal hyphae but not for roots and allows the separation between two plants. Three months after planting, an isotopic labeling experiment is conducted utilizing ^15^N. Plants are harvested 20 days after labeling. Percentage of root colonized by the inoculated *Funneliformis* strain and ^15^N abundance of taro plants are determined. **(B)** Pot culture experiments, consisting either to a model of monoculture (taro/taro) or a culture association (*P. officinalis*/taro). Plants are harvested after 12 weeks of growth and taro growth parameters are measured. The experiment aims the determination of the “competitor” status of *Funneliformis*. **(C)** Pot culture experiments, consisting in taro monoculture, to study the potential role of *Funneliformis* in plant pathogen defense. Plants are grown for 3 months to allow the establishment of *Funneliformis* and then are inoculated with a pathogen. Plants are harvested after 4 weeks of growth and growth parameters of both plants are measured. The experiment aims the determination the “ruderal” status of *Funneliformis*.

*Funneliformis* has been, however, described as a ruderal and stress tolerator taxa mainly involved in plant protection against biotic and abiotic stress rather than in plant nutrition (competitor) (Chagnon et al., 2013). Indeed, *Funneliformis* was shown to protect tropical plants against certain pathogens (Cardoso and Kuyper, 2006). Several strategies could be set up to experimentally test the significance of *Funneliformis* in nutrient and/or plant protection in systems associating *Pterocarpus-*taro. Compartmented microcosms with inoculated *Funneliformis* strains and the use of a ^15^N-labeled growth substrate as designed in Walder et al. (2012) could be used to demonstrate the N transfers through *Funneliformis* CMNs between the two studied plants (**Figure 3A**). Moreover the benefits of *Pterocarpus-*taro associations compared to taro monoculture could be estimated experimentally in pot cultures (**Figure 3B**), allowing the evaluation of *Funneliformis* contribution as competitor taxa (Chagnon et al., 2013). Finally, the introduction of a pathogen in inoculated or not inoculated pot cultures could be used to evaluate the significance of *Funneliformis* regarding plant protection (**Figure 3C**), but also the contribution of *Funneliformis* as ruderal taxa (Chagnon et al., 2013).

## Conclusion

Our study highlights the high AM fungal diversity and richness associated with roots of *Pterocarpus* (adult trees and seedlings) and taro. Although the AM fungal community is significantly different in terms of membership and structure between the types of plants, *Pterocarpus* and taro had few but predominant overlapping AM fungi, notably *Funneliformis* spp. (OTU1). From an agricultural point of view, in addition to the good tolerance of taro to waterlogging and shade under *Pterocarpus* swamp forests (Saur and Imbert, 2003), the preservation of *Pterocarpus* adult trees and their seedlings could be one of the main factors leading to high taro crop yields by maintaining N input on soil and a source of AM fungal inoculums that might form potential CMNs crucial for the establishment of taro.

## Data

Raw data are available under the BioProject ID PRJNA384862 (https://www.ncbi.nlm.nih.gov/bioproject).

## Author Contributions

AB and AnG designed the research and collected the samples. AlG and HS developed the methodology and performed statistical analyses. AlG and HS generated data. AB, AlG, and HS wrote the initial manuscript. AB, AlG, AnG, and HS contributed to the final manuscript. All the authors shared, edited and approved the final manuscript.

## Conflict of Interest Statement

The authors declare that the research was conducted in the absence of any commercial or financial relationships that could be construed as a potential conflict of interest.

## Footnotes

1 www.eurofinsgenomics.eu

2 http://www.mothur.org/wiki/454_SOP

## Supplementary Material

FIGURE S1 Map of Guadeloupe with the two sampling forest sites.

FIGURE S2 Non-metric multidimensional scaling (NMDS) ordination of *Pterocarpus* and taro root-associated AM fungal communities in Grande Ravine and Belle Plaine forest sites. Different colors represented the three types of plants.
