# Supplementary figures and images for: Molecular Characterization of Arbuscular Mycorrhizal Fungi in an Agroforestry System Reveals the Predominance of Funneliformis spp. Associated with Colocasia esculenta and Pterocarpus officinalis Adult Trees and Seedlings
Source: Front Microbiol. 2017 Jul 28;8:1426. doi: 10.3389/fmicb.2017.01426 (PMC5532380; doi:10.3389/fmicb.2017.01426)

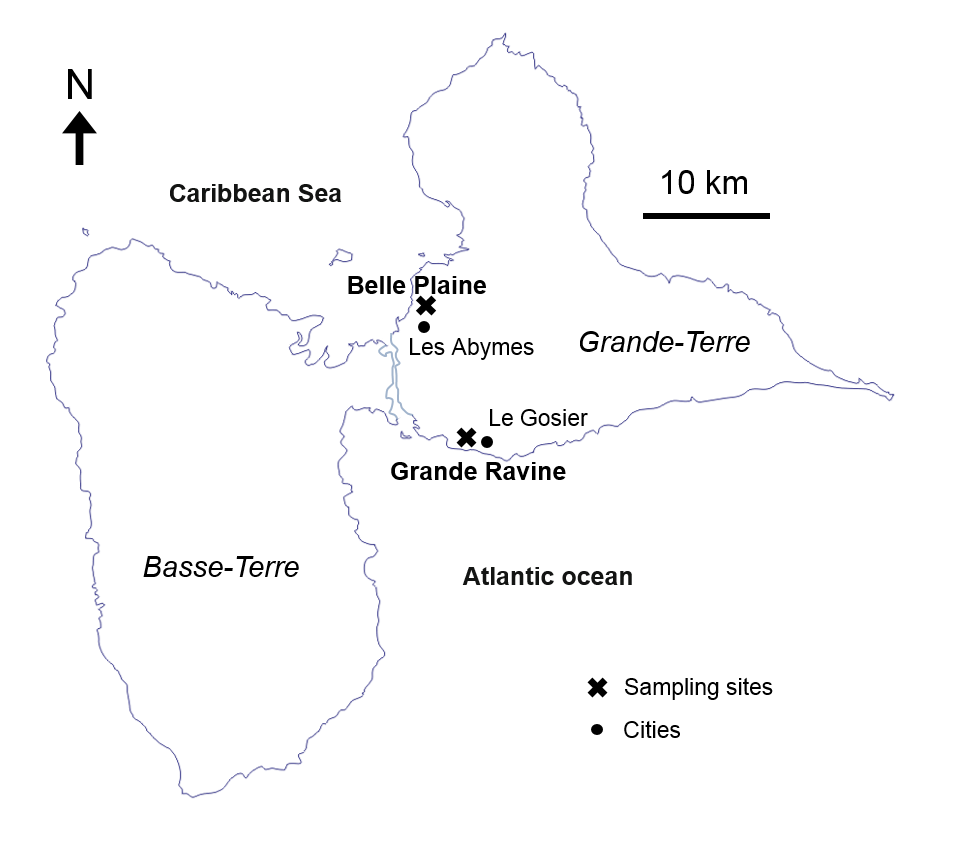

Supplement: FIGURE S1 — Map of Guadeloupe with the two sampling forest sites. [file Image_1.TIF]

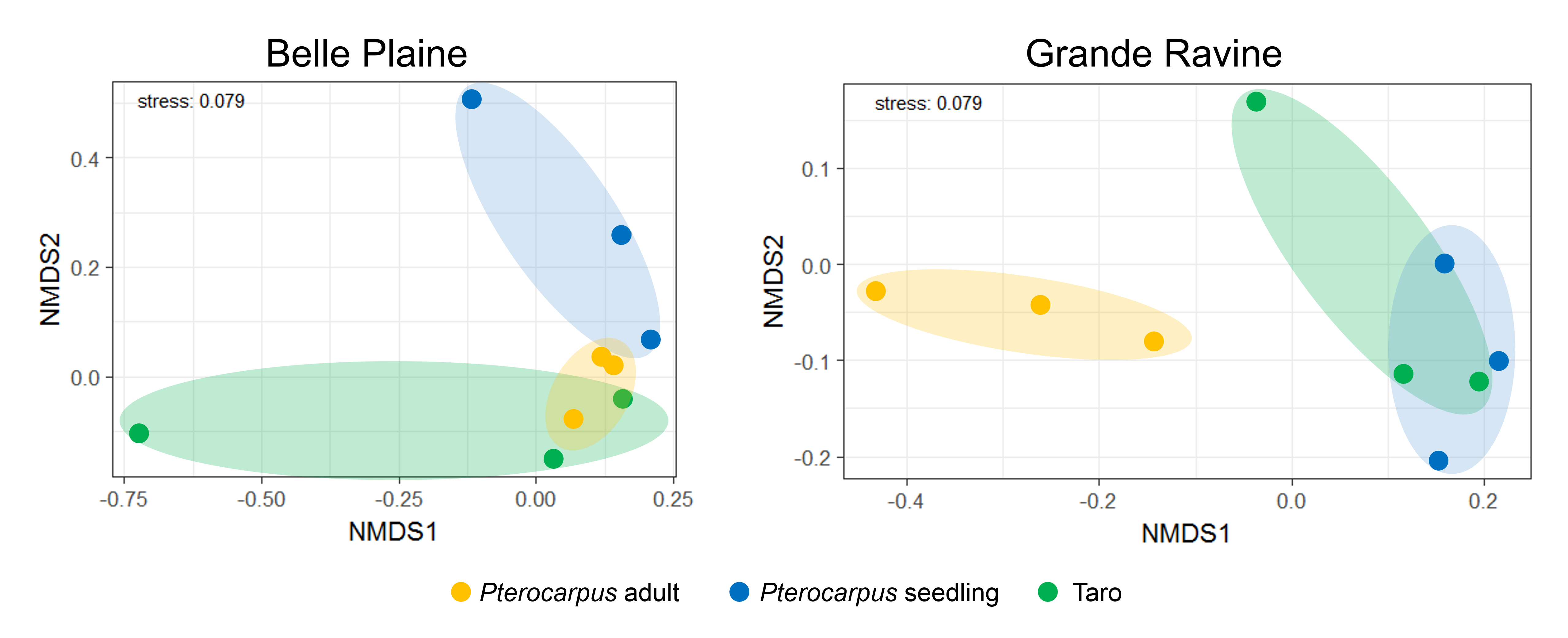

Supplement: FIGURE S2 — Non-metric multidimensional scaling (NMDS) ordination of Pterocarpus and taro root-associated AM fungal communities in Grande Ravine and Belle Plaine forest sites. Different colors represented the three types of plants. [file Image_2.TIF]
