# Supplementary material for: Molecular Characterization of Arbuscular Mycorrhizal Fungi in an Agroforestry System Reveals the Predominance of Funneliformis spp. Associated with Colocasia esculenta and Pterocarpus officinalis Adult Trees and Seedlings
Source: Front Microbiol. 2017 Jul 28;8:1426. doi: 10.3389/fmicb.2017.01426 (PMC5532380; doi:10.3389/fmicb.2017.01426)
Supplement: Supplementary file 3 [file Table_1.DOCX]

**Table S1. Soil characteristics of forest sites**

| Site | SOM ^1^ (g/kg) | N total (g/kg) | C/N | pH water | Ca total (g/kg) | CEC Metson (cmol+/kg) | P_2_0_5_ Olsen (g/kg) | K2O (g/kg) | MgO (g/kg) | Na_2_O (g/kg) |
| --- | --- | --- | --- | --- | --- | --- | --- | --- | --- | --- |
| Grande Ravine | 134.8 | 6 | 13.5 | 7.9 | 126 | 47 | 0.1 | 0.3 | 0.7 | 0.1 |
| Belle Plaine | 103.9 | 24 | 2.5 | 7.5 | 28 | 116 | 0.1 | 0.3 | 1.8 | 0.4 |

^1^ SOM, soil organic matter; N, nitrogen; C/N, carbon/nitrogen ratio; Ca, Calcium; CEC, cation exchange capacity; P_2_0_5_, phosphate; K_2_O, potassium; MgO, magnesium; Na_2_O, sodium
