# Supplementary material for: Molecular Characterization of Arbuscular Mycorrhizal Fungi in an Agroforestry System Reveals the Predominance of Funneliformis spp. Associated with Colocasia esculenta and Pterocarpus officinalis Adult Trees and Seedlings
Source: Front Microbiol. 2017 Jul 28;8:1426. doi: 10.3389/fmicb.2017.01426 (PMC5532380; doi:10.3389/fmicb.2017.01426)
Supplement: Supplementary file 4 [file Table_2.DOCX]

**Table S2**. Richness and diversity of AM fungal communities associated with taro and *Pterocarpus* in agroforestry systems.

| Locality | Plant type ^1^ | OTUs number (Richness) | Chao1  (Richness) | Shannon’s index  (diversity) | Inverse Simpson’s index (diversity) | Pielou’s index  (Evenness) | Good’s coverage ^2^ | Boneh  estimation |
| --- | --- | --- | --- | --- | --- | --- | --- | --- |
|  | P.a | 20±4 ^3^ | 32±9 | 1.28±0.24 | 2.58±0.33 | 0.24±0.04 | 97-98 % | 3±1 |
| Grande Ravine | P.s | 21±6 | 37±18 | 1.07±0.42 | 1.86±0.57 | 0.20±0.08 | 96-98 % | 3±1 |
|  | T. | 32±8 | 71±31 | 1.43±0.62 | 2.45±1.33 | 0.27±0.12 | 95-97 % | 6±1 |
|  | P.a | 26±7 | 63±23 | 1.21±0.20 | 1.94±0.18 | 0.23±0.04 | 95-97 % | 5±1 |
| Belle Plaine | P.s | 21±9 | 31±10 | 1.53±0.78 | 3.72±2.52 | 0.28±0.14 | 97 % | 3±1 |
|  | T. | 35±9 | 71±24 | 1.5±0.28 | 2.2±0.42 | 0.28±0.05 | 94-97 % | 6±2 |
|  |  |  |  |  |  |  |  |  |
| Locality | | ns^4^ | ns | ns | ns | ns |  |  |
| Plant type | | * | * | ns | ns | ns |  |  |
| Locality × plant type | | ns | ns | ns | ns | ns |  |  |

^1^ P.a, *Pterocarpus* adult tree; P.s, *Pterocarpus* seedlings; T., taro

^2^ Good’s coverage: sum of probabilities of observed classes calculated as (1 - (n/N)), where n is the number of singleton sequences and N is the total number of sequences

^3^ Values indicate mean ± standard deviation

^4^ Statistics were performed using PERMANOVA. Richness, diversity and evenness indexes were compared among plant type and locality, and the interaction between factors was also assessed. ‘*’ *P* < 0.05; ‘ns’ *P* > 0.05.
