# Supplementary material for: Molecular Characterization of Arbuscular Mycorrhizal Fungi in an Agroforestry System Reveals the Predominance of Funneliformis spp. Associated with Colocasia esculenta and Pterocarpus officinalis Adult Trees and Seedlings
Source: Front Microbiol. 2017 Jul 28;8:1426. doi: 10.3389/fmicb.2017.01426 (PMC5532380; doi:10.3389/fmicb.2017.01426)
Supplement: Supplementary file 5 [file Table_3.DOCX]

**Table S3**. Taxonomy assignment of 215 OTUs identified in *Pterocarpus*-Taro based agroforestry systems.

| OTU | Reads | Kingdom^1^ | Division | Class | Order | Family | Genus | Species |
| --- | --- | --- | --- | --- | --- | --- | --- | --- |
| Otu_1 | 4558 | Fungi_(100) | Glomeromycota_(100) | Glomeromycetes_(100) | Glomerales_(93) | Glomeraceae_(93) | Funneliformis_(88) | unclassified_(100) |
| Otu_2 | 1071 | Fungi_(100) | Glomeromycota_(100) | Glomeromycetes_(100) | Glomerales_(100) | Glomeraceae_(100) | Incertae_sedis_(99) | Glomus_indicum_(99) |
| Otu_3 | 722 | Fungi_(100) | Glomeromycota_(100) | Glomeromycetes_(100) | Diversisporales_(100) | Acaulosporaceae_(100) | Acaulospora_(100) | Acaulospora_longula_(100) |
| Otu_4 | 198 | Fungi_(100) | Glomeromycota_(100) | Glomeromycetes_(100) | Glomerales_(100) | Glomeraceae_(100) | Incertae_sedis_(53) | Glomus_indicum_(53) |
| Otu_5 | 170 | Fungi_(100) | Glomeromycota_(100) | Glomeromycetes_(100) | Glomerales_(100) | Glomeraceae_(100) | Funneliformis_(97) | unclassified_(100) |
| Otu_6 | 102 | Fungi_(100) | Glomeromycota_(100) | Glomeromycetes_(100) | Glomerales_(100) | Glomeraceae_(100) | Incertae_sedis_(100) | Glomus_indicum_(100) |
| Otu_7 | 59 | Fungi_(100) | Glomeromycota_(100) | Glomeromycetes_(100) | Diversisporales_(100) | Gigasporaceae_(100) | Gigaspora_(100) | Gigaspora_candida_(100) |
| Otu_8 | 70 | Fungi_(100) | Glomeromycota_(100) | Glomeromycetes_(100) | Glomerales_(99) | Glomeraceae_(99) | Rhizophagus_(99) | Rhizophagus_incertae_sedis_(99) |
| Otu_9 | 67 | Fungi_(100) | Glomeromycota_(100) | Glomeromycetes_(100) | Diversisporales_(100) | Gigasporaceae_(100) | Gigaspora_(99) | Gigaspora_albida_(99) |
| Otu_10 | 45 | Fungi_(100) | Glomeromycota_(100) | Glomeromycetes_(100) | Archaeosporales_(100) | Archaeosporaceae_(100) | Archaeospora_(100) | Archaeospora_schenckii_(100) |
| Otu_11 | 44 | Fungi_(100) | Glomeromycota_(100) | Glomeromycetes_(100) | Diversisporales_(100) | Gigasporaceae_(100) | Racocetra_(100) | Racocetra_fulgida_(100) |
| Otu_12 | 33 | Fungi_(100) | Glomeromycota_(100) | Glomeromycetes_(100) | Glomerales_(100) | Glomeraceae_(100) | Incertae_sedis_(88) | Glomus_indicum_(88) |
| Otu_13 | 32 | Fungi_(100) | Glomeromycota_(100) | Glomeromycetes_(100) | Diversisporales_(100) | Acaulosporaceae_(91) | Acaulospora_(91) | Acaulospora_longula_(91) |
| Otu_14 | 23 | Fungi_(100) | Glomeromycota_(100) | Glomeromycetes_(100) | Diversisporales_(100) | Gigasporaceae_(100) | Gigaspora_(100) | Gigaspora_candida_(100) |
| Otu_15 | 23 | Fungi_(100) | Glomeromycota_(100) | Glomeromycetes_(100) | Diversisporales_(100) | Gigasporaceae_(100) | Gigaspora_(100) | Gigaspora_candida_(100) |
| Otu_16 | 21 | Fungi_(100) | Glomeromycota_(100) | Glomeromycetes_(100) | Glomerales_(100) | Glomeraceae_(100) | Rhizophagus_(100) | Rhizophagus_vesiculiferus_(100) |
| Otu_17 | 21 | Fungi_(100) | Glomeromycota_(100) | Glomeromycetes_(100) | Diversisporales_(100) | Gigasporaceae_(100) | Gigaspora_(100) | Gigaspora_albida_(100) |
| Otu_18 | 19 | Fungi_(100) | Glomeromycota_(100) | Glomeromycetes_(100) | Archaeosporales_(100) | Geosiphonaceae_(100) | Geosiphon_(100) | Geosiphon_pyriformis_(100) |
| Otu_19 | 18 | Fungi_(100) | Glomeromycota_(100) | Glomeromycetes_(100) | Glomerales_(100) | Glomeraceae_(100) | Incertae_sedis_(84) | Glomus_indicum_(84) |
| Otu_20 | 18 | Fungi_(100) | Glomeromycota_(100) | Glomeromycetes_(100) | Glomerales_(100) | Glomeraceae_(100) | Rhizophagus_(100) | Rhizophagus_vesiculiferus_(100) |
| Otu_21 | 17 | Fungi_(100) | Glomeromycota_(100) | Glomeromycetes_(100) | Glomerales_(95) | Glomeraceae_(95) | Incertae_sedis_(89) | Glomus_indicum_(77) |
| Otu_22 | 17 | Fungi_(100) | Glomeromycota_(100) | Glomeromycetes_(100) | Diversisporales_(100) | Gigasporaceae_(100) | Gigaspora_(100) | Gigaspora_candida_(100) |
| Otu_23 | 17 | Fungi_(100) | Glomeromycota_(100) | Glomeromycetes_(100) | Glomerales_(95) | Glomeraceae_(95) | Rhizophagus_(95) | Rhizophagus_vesiculiferus_(95) |
| Otu_24 | 16 | Fungi_(100) | Glomeromycota_(100) | Glomeromycetes_(100) | Glomerales_(100) | Glomeraceae_(100) | Rhizophagus_(100) | Rhizophagus_incertae_sedis_(100) |
| Otu_25 | 15 | Fungi_(100) | Glomeromycota_(100) | Glomeromycetes_(100) | Glomerales_(100) | Glomeraceae_(100) | Incertae_sedis_(100) | Glomus_indicum_(100) |
| Otu_26 | 15 | Fungi_(100) | Glomeromycota_(100) | Glomeromycetes_(100) | Diversisporales_(100) | Gigasporaceae_(100) | Gigaspora_(100) | Gigaspora_candida_(100) |
| Otu_27 | 13 | Fungi_(100) | Glomeromycota_(100) | Glomeromycetes_(100) | Diversisporales_(100) | Gigasporaceae_(100) | Gigaspora_(93) | Gigaspora_albida_(93) |
| Otu_28 | 9 | Fungi_(100) | Glomeromycota_(100) | Glomeromycetes_(100) | Diversisporales_(100) | Gigasporaceae_(100) | Gigaspora_(100) | Gigaspora_candida_(100) |
| Otu_29 | 11 | Fungi_(100) | Glomeromycota_(100) | Glomeromycetes_(100) | Glomerales_(91) | Glomeraceae_(91) | Incertae_sedis_(73) | Glomus_indicum_(73) |
| Otu_30 | 10 | Fungi_(100) | Glomeromycota_(100) | Glomeromycetes_(100) | Diversisporales_(100) | Gigasporaceae_(100) | Gigaspora_(100) | Gigaspora_albida_(100) |
| Otu_31 | 10 | Fungi_(100) | Glomeromycota_(100) | Glomeromycetes_(100) | Glomerales_(90) | Glomeraceae_(90) | unclassified_(100) | unclassified_(100) |
| Otu_32 | 10 | Fungi_(100) | Glomeromycota_(100) | Glomeromycetes_(100) | Diversisporales_(100) | Gigasporaceae_(100) | Gigaspora_(100) | Gigaspora_albida_(100) |
| Otu_33 | 10 | Fungi_(100) | Glomeromycota_(100) | Glomeromycetes_(100) | Diversisporales_(100) | Gigasporaceae_(100) | Gigaspora_(100) | Gigaspora_candida_(100) |
| Otu_34 | 9 | Fungi_(100) | Glomeromycota_(100) | Glomeromycetes_(100) | Glomerales_(100) | Glomeraceae_(100) | Funneliformis_(100) | unclassified_(100) |
| Otu_35 | 8 | Fungi_(100) | Glomeromycota_(100) | Glomeromycetes_(100) | Diversisporales_(100) | Gigasporaceae_(100) | Gigaspora_(100) | Gigaspora_candida_(100) |
| Otu_36 | 8 | Fungi_(100) | Glomeromycota_(100) | Glomeromycetes_(100) | Glomerales_(100) | Glomeraceae_(100) | Funneliformis_(75) | Funneliformis_fragilistratus_(75) |
| Otu_37 | 7 | Fungi_(100) | Glomeromycota_(100) | Glomeromycetes_(100) | Glomerales_(100) | Glomeraceae_(100) | Rhizophagus_(100) | Rhizophagus_vesiculiferus_(100) |
| Otu_38 | 7 | Fungi_(100) | Glomeromycota_(100) | Glomeromycetes_(100) | Glomerales_(100) | Glomeraceae_(100) | Rhizophagus_(100) | Rhizophagus_vesiculiferus_(100) |
| Otu_39 | 7 | Fungi_(100) | Glomeromycota_(100) | Glomeromycetes_(100) | Diversisporales_(100) | Gigasporaceae_(100) | Gigaspora_(100) | Gigaspora_albida_(100) |
| Otu_40 | 7 | Fungi_(100) | Glomeromycota_(100) | Glomeromycetes_(100) | Diversisporales_(100) | Diversisporaceae_(100) | Diversispora_(86) | Diversispora_eburnea_(86) |
| Otu_41 | 7 | Fungi_(100) | Glomeromycota_(100) | Glomeromycetes_(100) | Glomerales_(100) | Glomeraceae_(100) | Incertae_sedis_(72) | Glomus_indicum_(72) |
| Otu_42 | 7 | Fungi_(100) | Glomeromycota_(100) | Glomeromycetes_(100) | Glomerales_(100) | Glomeraceae_(100) | Incertae_sedis_(100) | Glomus_indicum_(100) |
| Otu_43 | 6 | Fungi_(100) | Glomeromycota_(100) | Glomeromycetes_(100) | Diversisporales_(100) | Gigasporaceae_(100) | Gigaspora_(100) | Gigaspora_candida_(100) |
| Otu_44 | 6 | Fungi_(100) | Glomeromycota_(100) | Glomeromycetes_(100) | Glomerales_(100) | Glomeraceae_(100) | Incertae_sedis_(100) | Glomus_indicum_(100) |
| Otu_45 | 6 | Fungi_(100) | Glomeromycota_(100) | Glomeromycetes_(100) | Glomerales_(67) | Glomeraceae_(67) | unclassified_(100) | unclassified_(100) |
| Otu_46 | 6 | Fungi_(100) | Glomeromycota_(100) | Glomeromycetes_(100) | Glomerales_(100) | Glomeraceae_(100) | Incertae_sedis_(84) | Glomus_indicum_(84) |
| Otu_47 | 6 | Fungi_(100) | Glomeromycota_(100) | Glomeromycetes_(100) | Diversisporales_(100) | Gigasporaceae_(100) | Racocetra_(100) | Racocetra_fulgida_(100) |
| Otu_48 | 6 | Fungi_(100) | Glomeromycota_(100) | Glomeromycetes_(100) | Glomerales_(100) | Glomeraceae_(100) | Funneliformis_(100) | Funneliformis_fragilistratus_(67) |
| Otu_49 | 6 | Fungi_(100) | Glomeromycota_(100) | Glomeromycetes_(100) | Glomerales_(100) | Glomeraceae_(100) | Incertae_sedis_(100) | Glomus_indicum_(100) |
| Otu_50 | 5 | Fungi_(100) | Glomeromycota_(100) | Glomeromycetes_(100) | Diversisporales_(100) | Gigasporaceae_(100) | Gigaspora_(80) | Gigaspora_albida_(80) |
| Otu_51 | 5 | Fungi_(100) | Glomeromycota_(100) | Glomeromycetes_(100) | Glomerales_(80) | Glomeraceae_(80) | Funneliformis_(60) | Funneliformis_fragilistratus_(60) |
| Otu_52 | 5 | Fungi_(100) | Glomeromycota_(100) | Glomeromycetes_(100) | Archaeosporales_(100) | Archaeosporaceae_(100) | Archaeospora_(100) | Archaeospora_trappei_(100) |
| Otu_53 | 5 | Fungi_(100) | Glomeromycota_(100) | Glomeromycetes_(100) | Glomerales_(60) | Glomeraceae_(60) | Incertae_sedis_(60) | Glomus_indicum_(60) |
| Otu_54 | 4 | Fungi_(100) | Glomeromycota_(100) | Glomeromycetes_(100) | Archaeosporales_(100) | Archaeosporaceae_(100) | Archaeospora_(100) | Archaeospora_schenckii_(100) |
| Otu_55 | 4 | Fungi_(100) | Glomeromycota_(100) | Glomeromycetes_(100) | Diversisporales_(75) | Gigasporaceae_(75) | Racocetra_(75) | Racocetra_fulgida_(75) |
| Otu_56 | 4 | Fungi_(100) | Glomeromycota_(100) | Glomeromycetes_(100) | Glomerales_(100) | Glomeraceae_(100) | Rhizophagus_(100) | Rhizophagus_vesiculiferus_(100) |
| Otu_57 | 3 | Fungi_(100) | Glomeromycota_(100) | Glomeromycetes_(100) | Glomerales_(100) | Glomeraceae_(100) | Incertae_sedis_(100) | Glomus_indicum_(100) |
| Otu_58 | 4 | Fungi_(100) | Glomeromycota_(100) | Glomeromycetes_(100) | Diversisporales_(100) | Gigasporaceae_(100) | Gigaspora_(100) | Gigaspora_candida_(100) |
| Otu_59 | 4 | Fungi_(100) | Glomeromycota_(100) | Glomeromycetes_(100) | Glomerales_(100) | Glomeraceae_(100) | Funneliformis_(100) | Funneliformis_caledonius_(75) |
| Otu_61 | 4 | Fungi_(100) | Glomeromycota_(100) | Glomeromycetes_(100) | Glomerales_(100) | Glomeraceae_(100) | Funneliformis_(100) | Funneliformis_fragilistratus_(75) |
| Otu_62 | 4 | Fungi_(100) | Glomeromycota_(100) | Glomeromycetes_(100) | Diversisporales_(100) | Acaulosporaceae_(100) | Acaulospora_(100) | Acaulospora_longula_(100) |
| Otu_63 | 4 | Fungi_(100) | Glomeromycota_(100) | Glomeromycetes_(100) | unclassified_(100) | unclassified_(100) | unclassified_(100) | unclassified_(100) |
| Otu_64 | 3 | Fungi_(100) | Glomeromycota_(100) | Glomeromycetes_(100) | Glomerales_(100) | Glomeraceae_(100) | Funneliformis_(100) | Funneliformis_fragilistratus_(100) |
| Otu_65 | 4 | Fungi_(100) | Glomeromycota_(100) | Glomeromycetes_(100) | Glomerales_(100) | Glomeraceae_(100) | Rhizophagus_(100) | Rhizophagus_proliferus_(100) |
| Otu_66 | 4 | Fungi_(100) | Glomeromycota_(100) | Glomeromycetes_(100) | Glomerales_(100) | Glomeraceae_(100) | Funneliformis_(100) | Funneliformis_fragilistratus_(100) |
| Otu_67 | 3 | Fungi_(100) | Glomeromycota_(100) | Glomeromycetes_(100) | Diversisporales_(100) | Gigasporaceae_(100) | Racocetra_(100) | Racocetra_fulgida_(100) |
| Otu_68 | 3 | Fungi_(100) | Glomeromycota_(100) | Glomeromycetes_(100) | Archaeosporales_(100) | Archaeosporaceae_(100) | Archaeospora_(100) | Archaeospora_schenckii_(100) |
| Otu_69 | 3 | Fungi_(100) | Glomeromycota_(100) | Glomeromycetes_(100) | Glomerales_(100) | Glomeraceae_(100) | Incertae_sedis_(100) | Glomus_indicum_(100) |
| Otu_70 | 3 | Fungi_(100) | Glomeromycota_(100) | Glomeromycetes_(100) | Diversisporales_(67) | Gigasporaceae_(67) | Racocetra_(67) | Racocetra_fulgida_(67) |
| Otu_71 | 3 | Fungi_(100) | Glomeromycota_(100) | Glomeromycetes_(100) | Archaeosporales_(100) | Archaeosporaceae_(100) | Archaeospora_(100) | Archaeospora_schenckii_(100) |
| Otu_73 | 3 | Fungi_(100) | Glomeromycota_(100) | Glomeromycetes_(100) | Diversisporales_(100) | Gigasporaceae_(100) | Gigaspora_(100) | Gigaspora_candida_(100) |
| Otu_74 | 3 | Fungi_(100) | Glomeromycota_(100) | Glomeromycetes_(100) | Diversisporales_(100) | Gigasporaceae_(100) | Gigaspora_(100) | Gigaspora_incertae_sedis_(100) |
| Otu_75 | 3 | Fungi_(100) | Glomeromycota_(100) | Glomeromycetes_(100) | Glomerales_(100) | Glomeraceae_(100) | Rhizophagus_(100) | Rhizophagus_vesiculiferus_(100) |
| Otu_76 | 3 | Fungi_(100) | Glomeromycota_(100) | Glomeromycetes_(100) | Archaeosporales_(100) | Geosiphonaceae_(100) | Geosiphon_(100) | Geosiphon_pyriformis_(100) |
| Otu_78 | 3 | Fungi_(100) | Glomeromycota_(100) | Glomeromycetes_(100) | Glomerales_(67) | Glomeraceae_(67) | Funneliformis_(67) | unclassified_(100) |
| Otu_79 | 3 | Fungi_(100) | Glomeromycota_(100) | Glomeromycetes_(100) | Glomerales_(100) | Glomeraceae_(100) | Incertae_sedis_(100) | Glomus_indicum_(100) |
| Otu_80 | 3 | Fungi_(100) | Glomeromycota_(100) | Glomeromycetes_(100) | Glomerales_(100) | Glomeraceae_(100) | Funneliformis_(100) | Funneliformis_fragilistratus_(100) |
| Otu_81 | 3 | Fungi_(100) | Glomeromycota_(100) | Glomeromycetes_(100) | Glomerales_(100) | Glomeraceae_(100) | Incertae_sedis_(100) | Glomus_indicum_(100) |
| Otu_82 | 3 | Fungi_(100) | Glomeromycota_(100) | Glomeromycetes_(100) | Glomerales_(100) | Glomeraceae_(100) | Septoglomus_(100) | Septoglomus_constrictum_(100) |
| Otu_83 | 3 | Fungi_(100) | Glomeromycota_(100) | Glomeromycetes_(100) | Glomerales_(100) | Glomeraceae_(100) | Funneliformis_(100) | unclassified_(100) |
| Otu_84 | 3 | Fungi_(100) | Glomeromycota_(100) | Glomeromycetes_(100) | Diversisporales_(100) | Gigasporaceae_(100) | Racocetra_(100) | Racocetra_fulgida_(100) |
| Otu_85 | 2 | Fungi_(100) | Glomeromycota_(100) | Glomeromycetes_(100) | Diversisporales_(100) | Acaulosporaceae_(100) | Acaulospora_(100) | Acaulospora_longula_(100) |
| Otu_86 | 2 | Fungi_(100) | Glomeromycota_(100) | Glomeromycetes_(100) | Archaeosporales_(100) | Archaeosporaceae_(100) | Archaeospora_(100) | Archaeospora_trappei_(100) |
| Otu_87 | 2 | Fungi_(100) | Glomeromycota_(100) | Glomeromycetes_(100) | Glomerales_(100) | Glomeraceae_(100) | Funneliformis_(100) | Funneliformis_fragilistratus_(100) |
| Otu_88 | 2 | Fungi_(100) | Glomeromycota_(100) | Glomeromycetes_(100) | Diversisporales_(100) | Gigasporaceae_(100) | Racocetra_(100) | Racocetra_fulgida_(100) |
| Otu_89 | 2 | Fungi_(100) | Glomeromycota_(100) | Glomeromycetes_(100) | Diversisporales_(100) | Gigasporaceae_(100) | unclassified_(100) | unclassified_(100) |
| Otu_91 | 2 | Fungi_(100) | Glomeromycota_(100) | Glomeromycetes_(100) | Glomerales_(100) | Glomeraceae_(100) | Sclerocystis_(100) | Sclerocystis_sinuosa_(100) |
| Otu_93 | 2 | Fungi_(100) | Glomeromycota_(100) | Glomeromycetes_(100) | Diversisporales_(100) | Gigasporaceae_(100) | Gigaspora_(100) | Gigaspora_candida_(100) |
| Otu_94 | 2 | Fungi_(100) | Glomeromycota_(100) | Glomeromycetes_(100) | Diversisporales_(100) | Gigasporaceae_(100) | Gigaspora_(100) | Gigaspora_albida_(100) |
| Otu_95 | 2 | Fungi_(100) | Glomeromycota_(100) | Glomeromycetes_(100) | Glomerales_(100) | Glomeraceae_(100) | Incertae_sedis_(100) | Glomus_indicum_(100) |
| Otu_97 | 2 | Fungi_(100) | Glomeromycota_(100) | Glomeromycetes_(100) | Glomerales_(100) | Glomeraceae_(100) | Funneliformis_(100) | unclassified_(100) |
| Otu_98 | 2 | Fungi_(100) | Glomeromycota_(100) | Glomeromycetes_(100) | unclassified_(100) | unclassified_(100) | unclassified_(100) | unclassified_(100) |
| Otu_100 | 2 | Fungi_(100) | Glomeromycota_(100) | Glomeromycetes_(100) | Glomerales_(100) | Glomeraceae_(100) | unclassified_(100) | unclassified_(100) |
| Otu_101 | 2 | Fungi_(100) | Glomeromycota_(100) | Glomeromycetes_(100) | Glomerales_(100) | Glomeraceae_(100) | Funneliformis_(100) | unclassified_(100) |
| Otu_102 | 2 | Fungi_(100) | Glomeromycota_(100) | Glomeromycetes_(100) | Diversisporales_(100) | Gigasporaceae_(100) | Scutellospora_(100) | Scutellospora_projecturata_(100) |
| Otu_103 | 2 | Fungi_(100) | Glomeromycota_(100) | Glomeromycetes_(100) | Glomerales_(100) | Glomeraceae_(100) | Funneliformis_(100) | Funneliformis_fragilistratus_(100) |
| Otu_104 | 2 | Fungi_(100) | Glomeromycota_(100) | Glomeromycetes_(100) | Diversisporales_(100) | Gigasporaceae_(100) | Racocetra_(100) | Racocetra_fulgida_(100) |
| Otu_105 | 2 | Fungi_(100) | Glomeromycota_(100) | Glomeromycetes_(100) | Glomerales_(100) | Glomeraceae_(100) | Incertae_sedis_(100) | Glomus_indicum_(100) |
| Otu_106 | 2 | Fungi_(100) | Glomeromycota_(100) | Glomeromycetes_(100) | Diversisporales_(100) | Gigasporaceae_(100) | Gigaspora_(100) | Gigaspora_candida_(100) |
| Otu_107 | 2 | Fungi_(100) | Glomeromycota_(100) | Glomeromycetes_(100) | Glomerales_(100) | Glomeraceae_(100) | Rhizophagus_(100) | Rhizophagus_vesiculiferus_(100) |
| Otu_108 | 2 | Fungi_(100) | Glomeromycota_(100) | Glomeromycetes_(100) | Glomerales_(100) | Glomeraceae_(100) | Rhizophagus_(100) | Rhizophagus_vesiculiferus_(100) |
| Otu_109 | 2 | Fungi_(100) | Glomeromycota_(100) | Glomeromycetes_(100) | Glomerales_(100) | Glomeraceae_(100) | unclassified_(100) | unclassified_(100) |
| Otu_110 | 2 | Fungi_(100) | Glomeromycota_(100) | Glomeromycetes_(100) | Glomerales_(100) | Glomeraceae_(100) | Funneliformis_(100) | Funneliformis_fragilistratus_(100) |
| Otu_111 | 2 | Fungi_(100) | Glomeromycota_(100) | Glomeromycetes_(100) | Glomerales_(100) | Glomeraceae_(100) | Rhizophagus_(100) | Rhizophagus_vesiculiferus_(100) |
| Otu_112 | 2 | Fungi_(100) | Glomeromycota_(100) | Glomeromycetes_(100) | Glomerales_(100) | Glomeraceae_(100) | Incertae_sedis_(100) | Glomus_indicum_(100) |
| Otu_115 | 2 | Fungi_(100) | Glomeromycota_(100) | Glomeromycetes_(100) | Glomerales_(100) | Glomeraceae_(100) | Incertae_sedis_(100) | Glomus_indicum_(100) |
| Otu_116 | 2 | Fungi_(100) | Glomeromycota_(100) | Glomeromycetes_(100) | Archaeosporales_(100) | Archaeosporaceae_(100) | Archaeospora_(100) | Archaeospora_trappei_(100) |
| Otu_119 | 2 | Fungi_(100) | Glomeromycota_(100) | Glomeromycetes_(100) | unclassified_(100) | unclassified_(100) | unclassified_(100) | unclassified_(100) |
| Otu_120 | 2 | Fungi_(100) | Glomeromycota_(100) | Glomeromycetes_(100) | Glomerales_(100) | Glomeraceae_(100) | Rhizophagus_(100) | Rhizophagus_incertae_sedis_(100) |
| Otu_121 | 2 | Fungi_(100) | Glomeromycota_(100) | Glomeromycetes_(100) | Glomerales_(100) | Glomeraceae_(100) | Incertae_sedis_(100) | Glomus_indicum_(100) |
| Otu_123 | 2 | Fungi_(100) | Glomeromycota_(100) | Glomeromycetes_(100) | unclassified_(100) | unclassified_(100) | unclassified_(100) | unclassified_(100) |
| Otu_124 | 2 | Fungi_(100) | Glomeromycota_(100) | Glomeromycetes_(100) | Diversisporales_(100) | Gigasporaceae_(100) | Scutellospora_(100) | Scutellospora_projecturata_(100) |
| Otu_125 | 2 | Fungi_(100) | Glomeromycota_(100) | Glomeromycetes_(100) | Diversisporales_(100) | Gigasporaceae_(100) | Gigaspora_(100) | Gigaspora_candida_(100) |
| Otu_127 | 2 | Fungi_(100) | Glomeromycota_(100) | Glomeromycetes_(100) | unclassified_(100) | unclassified_(100) | unclassified_(100) | unclassified_(100) |
| Otu_129 | 2 | Fungi_(100) | Glomeromycota_(100) | Glomeromycetes_(100) | Diversisporales_(100) | Gigasporaceae_(100) | Gigaspora_(100) | Gigaspora_candida_(100) |
| Otu_130 | 2 | Fungi_(100) | Glomeromycota_(100) | Glomeromycetes_(100) | Glomerales_(100) | Glomeraceae_(100) | Rhizophagus_(100) | Rhizophagus_intraradices_(100) |
| Otu_132 | 2 | Fungi_(100) | Glomeromycota_(100) | Glomeromycetes_(100) | Glomerales_(100) | Glomeraceae_(100) | Rhizophagus_(100) | Rhizophagus_vesiculiferus_(100) |
| Otu_133 | 2 | Fungi_(100) | Glomeromycota_(100) | Glomeromycetes_(100) | Glomerales_(100) | Glomeraceae_(100) | Rhizophagus_(100) | Rhizophagus_vesiculiferus_(100) |
| Otu_134 | 2 | Fungi_(100) | Glomeromycota_(100) | Glomeromycetes_(100) | Glomerales_(100) | Glomeraceae_(100) | Rhizophagus_(100) | Rhizophagus_incertae_sedis_(100) |
| Otu_135 | 2 | Fungi_(100) | Glomeromycota_(100) | Glomeromycetes_(100) | Glomerales_(100) | Glomeraceae_(100) | Rhizophagus_(100) | Rhizophagus_vesiculiferus_(100) |
| Otu_136 | 2 | Fungi_(100) | Glomeromycota_(100) | Glomeromycetes_(100) | Paraglomerales_(100) | Paraglomeraceae_(100) | Paraglomus_(100) | Paraglomus_occultum_(100) |
| Otu_137 | 2 | Fungi_(100) | Glomeromycota_(100) | Glomeromycetes_(100) | Glomerales_(100) | Glomeraceae_(100) | Funneliformis_(100) | Funneliformis_fragilistratus_(100) |
| Otu_138 | 2 | Fungi_(100) | Glomeromycota_(100) | Glomeromycetes_(100) | Glomerales_(100) | Glomeraceae_(100) | Incertae_sedis_(100) | Glomus_indicum_(100) |
| Otu_139 | 1 | Fungi_(100) | Glomeromycota_(100) | Glomeromycetes_(100) | Glomerales_(100) | Glomeraceae_(100) | Funneliformis_(100) | Funneliformis_fragilistratus_(100) |
| Otu_140 | 1 | Fungi_(100) | Glomeromycota_(100) | Glomeromycetes_(100) | Glomerales_(100) | Glomeraceae_(100) | Incertae_sedis_(100) | Glomus_indicum_(100) |
| Otu_141 | 1 | Fungi_(100) | Glomeromycota_(100) | Glomeromycetes_(100) | Diversisporales_(100) | Diversisporaceae_(100) | Diversispora_(100) | Diversispora_eburnea_(100) |
| Otu_144 | 1 | Fungi_(100) | Glomeromycota_(100) | Glomeromycetes_(100) | Glomerales_(100) | Glomeraceae_(100) | Funneliformis_(100) | Funneliformis_fragilistratus_(100) |
| Otu_145 | 1 | Fungi_(100) | Glomeromycota_(100) | Glomeromycetes_(100) | Glomerales_(100) | Glomeraceae_(100) | Incertae_sedis_(100) | Glomus_indicum_(100) |
| Otu_146 | 1 | Fungi_(100) | Glomeromycota_(100) | Glomeromycetes_(100) | Glomerales_(100) | Glomeraceae_(100) | Incertae_sedis_(100) | Glomus_indicum_(100) |
| Otu_148 | 1 | Fungi_(100) | Glomeromycota_(100) | Glomeromycetes_(100) | Diversisporales_(100) | Gigasporaceae_(100) | Scutellospora_(100) | Scutellospora_projecturata_(100) |
| Otu_152 | 1 | Fungi_(100) | Glomeromycota_(100) | Glomeromycetes_(100) | Glomerales_(100) | Glomeraceae_(100) | Glomus_(100) | Glomus_incertae_sedis_(100) |
| Otu_153 | 1 | Fungi_(100) | Glomeromycota_(100) | Glomeromycetes_(100) | Diversisporales_(100) | Gigasporaceae_(100) | Gigaspora_(100) | Gigaspora_candida_(100) |
| Otu_154 | 1 | Fungi_(100) | Glomeromycota_(100) | Glomeromycetes_(100) | Glomerales_(100) | Glomeraceae_(100) | Funneliformis_(100) | Funneliformis_caledonius_(100) |
| Otu_155 | 1 | Fungi_(100) | Glomeromycota_(100) | Glomeromycetes_(100) | Diversisporales_(100) | Gigasporaceae_(100) | Cetraspora_(100) | Cetraspora_nodosa_(100) |
| Otu_158 | 1 | Fungi_(100) | Glomeromycota_(100) | Glomeromycetes_(100) | Glomerales_(100) | Glomeraceae_(100) | Sclerocystis_(100) | Sclerocystis_sinuosa_(100) |
| Otu_160 | 1 | Fungi_(100) | Glomeromycota_(100) | Glomeromycetes_(100) | Glomerales_(100) | Glomeraceae_(100) | Funneliformis_(100) | Funneliformis_fragilistratus_(100) |
| Otu_163 | 1 | Fungi_(100) | Glomeromycota_(100) | Glomeromycetes_(100) | Glomerales_(100) | Glomeraceae_(100) | Funneliformis_(100) | Funneliformis_fragilistratus_(100) |
| Otu_164 | 1 | Fungi_(100) | Glomeromycota_(100) | Glomeromycetes_(100) | Diversisporales_(100) | Gigasporaceae_(100) | Gigaspora_(100) | Gigaspora_candida_(100) |
| Otu_165 | 1 | Fungi_(100) | Glomeromycota_(100) | Glomeromycetes_(100) | Diversisporales_(100) | Gigasporaceae_(100) | Racocetra_(100) | Racocetra_fulgida_(100) |
| Otu_167 | 1 | Fungi_(100) | Glomeromycota_(100) | Glomeromycetes_(100) | Glomerales_(100) | Glomeraceae_(100) | Funneliformis_(100) | Funneliformis_fragilistratus_(100) |
| Otu_169 | 1 | Fungi_(100) | Glomeromycota_(100) | Glomeromycetes_(100) | Diversisporales_(100) | Gigasporaceae_(100) | Racocetra_(100) | Racocetra_fulgida_(100) |
| Otu_173 | 1 | Fungi_(100) | Glomeromycota_(100) | Glomeromycetes_(100) | Diversisporales_(100) | Gigasporaceae_(100) | Scutellospora_(100) | Scutellospora_projecturata_(100) |
| Otu_174 | 1 | Fungi_(100) | Glomeromycota_(100) | Glomeromycetes_(100) | Glomerales_(100) | Glomeraceae_(100) | Funneliformis_(100) | Funneliformis_fragilistratus_(100) |
| Otu_175 | 1 | Fungi_(100) | Glomeromycota_(100) | Glomeromycetes_(100) | Glomerales_(100) | Glomeraceae_(100) | Sclerocystis_(100) | Sclerocystis_sinuosa_(100) |
| Otu_177 | 1 | Fungi_(100) | Glomeromycota_(100) | Glomeromycetes_(100) | Glomerales_(100) | Glomeraceae_(100) | Rhizophagus_(100) | Rhizophagus_manihotis_(100) |
| Otu_179 | 1 | Fungi_(100) | Glomeromycota_(100) | Glomeromycetes_(100) | Diversisporales_(100) | Gigasporaceae_(100) | Racocetra_(100) | Racocetra_fulgida_(100) |
| Otu_180 | 1 | Fungi_(100) | Glomeromycota_(100) | Glomeromycetes_(100) | Diversisporales_(100) | Gigasporaceae_(100) | Scutellospora_(100) | Scutellospora_projecturata_(100) |
| Otu_181 | 1 | Fungi_(100) | Glomeromycota_(100) | Glomeromycetes_(100) | Archaeosporales_(100) | Archaeosporaceae_(100) | Archaeospora_(100) | Archaeospora_schenckii_(100) |
| Otu_182 | 1 | Fungi_(100) | Glomeromycota_(100) | Glomeromycetes_(100) | Glomerales_(100) | Glomeraceae_(100) | Funneliformis_(100) | Funneliformis_fragilistratus_(100) |
| Otu_183 | 1 | Fungi_(100) | Glomeromycota_(100) | Glomeromycetes_(100) | Glomerales_(100) | Glomeraceae_(100) | Incertae_sedis_(100) | Glomus_indicum_(100) |
| Otu_184 | 1 | Fungi_(100) | Glomeromycota_(100) | Glomeromycetes_(100) | Glomerales_(100) | Glomeraceae_(100) | Funneliformis_(100) | Funneliformis_fragilistratus_(100) |
| Otu_189 | 1 | Fungi_(100) | Glomeromycota_(100) | Glomeromycetes_(100) | Diversisporales_(100) | Pacisporaceae_(100) | Pacispora_(100) | Pacispora_franciscana_(100) |
| Otu_191 | 1 | Fungi_(100) | Glomeromycota_(100) | Glomeromycetes_(100) | Diversisporales_(100) | Gigasporaceae_(100) | Cetraspora_(100) | Cetraspora_pellucida_(100) |
| Otu_192 | 1 | Fungi_(100) | Glomeromycota_(100) | Glomeromycetes_(100) | Glomerales_(100) | Glomeraceae_(100) | Incertae_sedis_(100) | Glomus_indicum_(100) |
| Otu_200 | 1 | Fungi_(100) | Glomeromycota_(100) | Glomeromycetes_(100) | Diversisporales_(100) | Gigasporaceae_(100) | Racocetra_(100) | Racocetra_fulgida_(100) |
| Otu_201 | 1 | Fungi_(100) | Glomeromycota_(100) | Glomeromycetes_(100) | Glomerales_(100) | Glomeraceae_(100) | Funneliformis_(100) | Funneliformis_fragilistratus_(100) |
| Otu_203 | 1 | Fungi_(100) | Glomeromycota_(100) | Glomeromycetes_(100) | Glomerales_(100) | Glomeraceae_(100) | Glomus_(100) | Glomus_incertae_sedis_(100) |
| Otu_204 | 1 | Fungi_(100) | Glomeromycota_(100) | Glomeromycetes_(100) | Diversisporales_(100) | Gigasporaceae_(100) | Gigaspora_(100) | Gigaspora_candida_(100) |
| Otu_205 | 1 | Fungi_(100) | Glomeromycota_(100) | Glomeromycetes_(100) | Glomerales_(100) | Glomeraceae_(100) | Incertae_sedis_(100) | Glomus_indicum_(100) |
| Otu_207 | 1 | Fungi_(100) | Glomeromycota_(100) | Glomeromycetes_(100) | Diversisporales_(100) | Gigasporaceae_(100) | Cetraspora_(100) | Cetraspora_nodosa_(100) |
| Otu_208 | 1 | Fungi_(100) | Glomeromycota_(100) | Glomeromycetes_(100) | Diversisporales_(100) | Acaulosporaceae_(100) | Acaulospora_(100) | Acaulospora_longula_(100) |
| Otu_212 | 1 | Fungi_(100) | Glomeromycota_(100) | Glomeromycetes_(100) | Glomerales_(100) | Glomeraceae_(100) | Rhizophagus_(100) | Rhizophagus_proliferus_(100) |
| Otu_213 | 1 | Fungi_(100) | Glomeromycota_(100) | Glomeromycetes_(100) | Glomerales_(100) | Glomeraceae_(100) | Funneliformis_(100) | Funneliformis_fragilistratus_(100) |
| Otu_214 | 1 | Fungi_(100) | Glomeromycota_(100) | Glomeromycetes_(100) | Glomerales_(100) | Glomeraceae_(100) | Incertae_sedis_(100) | Glomus_indicum_(100) |
| Otu_215 | 1 | Fungi_(100) | Glomeromycota_(100) | Glomeromycetes_(100) | Diversisporales_(100) | Gigasporaceae_(100) | Gigaspora_(100) | Gigaspora_candida_(100) |
| Otu_218 | 1 | Fungi_(100) | Glomeromycota_(100) | Glomeromycetes_(100) | Glomerales_(100) | Glomeraceae_(100) | Rhizophagus_(100) | Rhizophagus_incertae_sedis_(100) |
| Otu_224 | 1 | Fungi_(100) | Glomeromycota_(100) | Glomeromycetes_(100) | Glomerales_(100) | Glomeraceae_(100) | Rhizophagus_(100) | Rhizophagus_vesiculiferus_(100) |
| Otu_226 | 1 | Fungi_(100) | Glomeromycota_(100) | Glomeromycetes_(100) | Diversisporales_(100) | Gigasporaceae_(100) | Cetraspora_(100) | Cetraspora_nodosa_(100) |
| Otu_228 | 1 | Fungi_(100) | Glomeromycota_(100) | Glomeromycetes_(100) | Glomerales_(100) | Glomeraceae_(100) | Rhizophagus_(100) | Rhizophagus_incertae_sedis_(100) |
| Otu_234 | 1 | Fungi_(100) | Glomeromycota_(100) | Glomeromycetes_(100) | Glomerales_(100) | Glomeraceae_(100) | Sclerocystis_(100) | Sclerocystis_sinuosa_(100) |
| Otu_235 | 1 | Fungi_(100) | Glomeromycota_(100) | Glomeromycetes_(100) | Diversisporales_(100) | Acaulosporaceae_(100) | Acaulospora_(100) | Acaulospora_longula_(100) |
| Otu_236 | 1 | Fungi_(100) | Glomeromycota_(100) | Glomeromycetes_(100) | Glomerales_(100) | Glomeraceae_(100) | Sclerocystis_(100) | Sclerocystis_sinuosa_(100) |
| Otu_237 | 1 | Fungi_(100) | Glomeromycota_(100) | Glomeromycetes_(100) | Glomerales_(100) | Glomeraceae_(100) | Rhizophagus_(100) | Rhizophagus_incertae_sedis_(100) |
| Otu_238 | 1 | Fungi_(100) | Glomeromycota_(100) | Glomeromycetes_(100) | Diversisporales_(100) | Diversisporaceae_(100) | Diversispora_(100) | Diversispora_eburnea_(100) |
| Otu_239 | 1 | Fungi_(100) | Glomeromycota_(100) | Glomeromycetes_(100) | Glomerales_(100) | Glomeraceae_(100) | Funneliformis_(100) | Funneliformis_caledonius_(100) |
| Otu_240 | 1 | Fungi_(100) | Glomeromycota_(100) | Glomeromycetes_(100) | Glomerales_(100) | Glomeraceae_(100) | Funneliformis_(100) | Funneliformis_fragilistratus_(100) |
| Otu_241 | 1 | Fungi_(100) | Glomeromycota_(100) | Glomeromycetes_(100) | Glomerales_(100) | Glomeraceae_(100) | Funneliformis_(100) | Funneliformis_fragilistratus_(100) |
| Otu_247 | 1 | Fungi_(100) | Glomeromycota_(100) | Glomeromycetes_(100) | Diversisporales_(100) | Gigasporaceae_(100) | Racocetra_(100) | Racocetra_fulgida_(100) |
| Otu_248 | 1 | Fungi_(100) | Glomeromycota_(100) | Glomeromycetes_(100) | Diversisporales_(100) | Acaulosporaceae_(100) | Acaulospora_(100) | Acaulospora_longula_(100) |
| Otu_251 | 1 | Fungi_(100) | Glomeromycota_(100) | Glomeromycetes_(100) | Glomerales_(100) | Glomeraceae_(100) | Rhizophagus_(100) | Rhizophagus_incertae_sedis_(100) |
| Otu_254 | 1 | Fungi_(100) | Glomeromycota_(100) | Glomeromycetes_(100) | Diversisporales_(100) | Gigasporaceae_(100) | Cetraspora_(100) | Cetraspora_nodosa_(100) |
| Otu_264 | 1 | Fungi_(100) | Glomeromycota_(100) | Glomeromycetes_(100) | Glomerales_(100) | Glomeraceae_(100) | Rhizophagus_(100) | Rhizophagus_incertae_sedis_(100) |
| Otu_265 | 1 | Fungi_(100) | Glomeromycota_(100) | Glomeromycetes_(100) | Paraglomerales_(100) | Paraglomeraceae_(100) | Paraglomus_(100) | Paraglomus_brasilianum_(100) |
| Otu_266 | 1 | Fungi_(100) | Glomeromycota_(100) | Glomeromycetes_(100) | Paraglomerales_(100) | Paraglomeraceae_(100) | Paraglomus_(100) | Paraglomus_brasilianum_(100) |
| Otu_267 | 1 | Fungi_(100) | Glomeromycota_(100) | Glomeromycetes_(100) | Diversisporales_(100) | Pacisporaceae_(100) | Pacispora_(100) | Pacispora_franciscana_(100) |
| Otu_268 | 1 | Fungi_(100) | Glomeromycota_(100) | Glomeromycetes_(100) | Glomerales_(100) | Glomeraceae_(100) | Funneliformis_(100) | Funneliformis_fragilistratus_(100) |
| Otu_269 | 1 | Fungi_(100) | Glomeromycota_(100) | Glomeromycetes_(100) | Diversisporales_(100) | Gigasporaceae_(100) | Gigaspora_(100) | Gigaspora_candida_(100) |
| Otu_272 | 1 | Fungi_(100) | Glomeromycota_(100) | Glomeromycetes_(100) | Glomerales_(100) | Glomeraceae_(100) | Funneliformis_(100) | Funneliformis_fragilistratus_(100) |
| Otu_273 | 1 | Fungi_(100) | Glomeromycota_(100) | Glomeromycetes_(100) | Glomerales_(100) | Glomeraceae_(100) | Incertae_sedis_(100) | Glomus_indicum_(100) |
| Otu_274 | 1 | Fungi_(100) | Glomeromycota_(100) | Glomeromycetes_(100) | Diversisporales_(100) | Gigasporaceae_(100) | Gigaspora_(100) | Gigaspora_candida_(100) |
| Otu_277 | 1 | Fungi_(100) | Glomeromycota_(100) | Glomeromycetes_(100) | Diversisporales_(100) | Gigasporaceae_(100) | Gigaspora_(100) | Gigaspora_candida_(100) |
| Otu_279 | 1 | Fungi_(100) | Glomeromycota_(100) | Glomeromycetes_(100) | Diversisporales_(100) | Gigasporaceae_(100) | Gigaspora_(100) | Gigaspora_albida_(100) |
| Otu_280 | 1 | Fungi_(100) | Glomeromycota_(100) | Glomeromycetes_(100) | Diversisporales_(100) | Gigasporaceae_(100) | Cetraspora_(100) | Cetraspora_nodosa_(100) |
| Otu_281 | 1 | Fungi_(100) | Glomeromycota_(100) | Glomeromycetes_(100) | Glomerales_(100) | Glomeraceae_(100) | Incertae_sedis_(100) | Glomus_indicum_(100) |
| Otu_282 | 1 | Fungi_(100) | Glomeromycota_(100) | Glomeromycetes_(100) | Glomerales_(100) | Glomeraceae_(100) | Incertae_sedis_(100) | Glomus_indicum_(100) |
| Otu_283 | 1 | Fungi_(100) | Glomeromycota_(100) | Glomeromycetes_(100) | Archaeosporales_(100) | Archaeosporaceae_(100) | Archaeospora_(100) | Archaeospora_schenckii_(100) |
| Otu_285 | 1 | Fungi_(100) | Glomeromycota_(100) | Glomeromycetes_(100) | Glomerales_(100) | Glomeraceae_(100) | Rhizophagus_(100) | Rhizophagus_incertae_sedis_(100) |
| Otu_286 | 1 | Fungi_(100) | Glomeromycota_(100) | Glomeromycetes_(100) | Glomerales_(100) | Glomeraceae_(100) | Rhizophagus_(100) | Rhizophagus_incertae_sedis_(100) |
| Otu_287 | 1 | Fungi_(100) | Glomeromycota_(100) | Glomeromycetes_(100) | Diversisporales_(100) | Gigasporaceae_(100) | Scutellospora_(100) | Scutellospora_projecturata_(100) |
| Otu_289 | 1 | Fungi_(100) | Glomeromycota_(100) | Glomeromycetes_(100) | Glomerales_(100) | Glomeraceae_(100) | Sclerocystis_(100) | Sclerocystis_sinuosa_(100) |
| Otu_294 | 1 | Fungi_(100) | Glomeromycota_(100) | Glomeromycetes_(100) | Glomerales_(100) | Glomeraceae_(100) | Funneliformis_(100) | Funneliformis_fragilistratus_(100) |
| Otu_296 | 1 | Fungi_(100) | Glomeromycota_(100) | Glomeromycetes_(100) | Archaeosporales_(100) | Archaeosporaceae_(100) | Archaeospora_(100) | Archaeospora_schenckii_(100) |
| Otu_299 | 1 | Fungi_(100) | Glomeromycota_(100) | Glomeromycetes_(100) | Archaeosporales_(100) | Archaeosporaceae_(100) | Archaeospora_(100) | Archaeospora_trappei_(100) |
| Otu_302 | 1 | Fungi_(100) | Glomeromycota_(100) | Glomeromycetes_(100) | Glomerales_(100) | Glomeraceae_(100) | Funneliformis_(100) | Funneliformis_caledonius_(100) |
| Otu_307 | 1 | Fungi_(100) | Glomeromycota_(100) | Glomeromycetes_(100) | Glomerales_(100) | Glomeraceae_(100) | Incertae_sedis_(100) | Glomus_indicum_(100) |
| Otu_308 | 1 | Fungi_(100) | Glomeromycota_(100) | Glomeromycetes_(100) | Glomerales_(100) | Glomeraceae_(100) | Funneliformis_(100) | Funneliformis_fragilistratus_(100) |
| Otu_310 | 1 | Fungi_(100) | Glomeromycota_(100) | Glomeromycetes_(100) | Glomerales_(100) | Glomeraceae_(100) | Funneliformis_(100) | Funneliformis_caledonius_(100) |
| Otu_313 | 1 | Fungi_(100) | Glomeromycota_(100) | Glomeromycetes_(100) | Diversisporales_(100) | Gigasporaceae_(100) | Scutellospora_(100) | Scutellospora_projecturata_(100) |
| Otu_314 | 1 | Fungi_(100) | Glomeromycota_(100) | Glomeromycetes_(100) | Glomerales_(100) | Glomeraceae_(100) | Incertae_sedis_(100) | Glomus_indicum_(100) |
| Otu_316 | 1 | Fungi_(100) | Glomeromycota_(100) | Glomeromycetes_(100) | Glomerales_(100) | Glomeraceae_(100) | Incertae_sedis_(100) | Glomus_indicum_(100) |
| Otu_317 | 1 | Fungi_(100) | Glomeromycota_(100) | Glomeromycetes_(100) | Glomerales_(100) | Glomeraceae_(100) | Incertae_sedis_(100) | Glomus_indicum_(100) |
| Otu_318 | 1 | Fungi_(100) | Glomeromycota_(100) | Glomeromycetes_(100) | Glomerales_(100) | Glomeraceae_(100) | Incertae_sedis_(100) | Glomus_indicum_(100) |
| Otu_319 | 1 | Fungi_(100) | Glomeromycota_(100) | Glomeromycetes_(100) | Glomerales_(100) | Glomeraceae_(100) | Funneliformis_(100) | Funneliformis_caledonius_(100) |
| Otu_320 | 1 | Fungi_(100) | Glomeromycota_(100) | Glomeromycetes_(100) | Glomerales_(100) | Glomeraceae_(100) | Funneliformis_(100) | Funneliformis_fragilistratus_(100) |
| Otu_322 | 1 | Fungi_(100) | Glomeromycota_(100) | Glomeromycetes_(100) | Diversisporales_(100) | Gigasporaceae_(100) | Gigaspora_(100) | Gigaspora_candida_(100) |

^1^ The number between () indicate the confidence threshold for the OTU assignment.
