# Supplementary material for: Molecular Characterization of Arbuscular Mycorrhizal Fungi in an Agroforestry System Reveals the Predominance of Funneliformis spp. Associated with Colocasia esculenta and Pterocarpus officinalis Adult Trees and Seedlings
Source: Front Microbiol. 2017 Jul 28;8:1426. doi: 10.3389/fmicb.2017.01426 (PMC5532380; doi:10.3389/fmicb.2017.01426)
Supplement: Supplementary file 6 [file Table_4.DOCX]

**Table S4**. Comparison of AM fungal OTU abundances between localities (Grande Ravine and Belle Plaine).

|  | Grande Ravine | | Belle Plaine | |  |
| --- | --- | --- | --- | --- | --- |
| OTU ^1^ | reads | frequency | reads | frequency | *P*-value ^2^ |
| Otu_1 | 2422 | 1 | 2137 | 1 | 0.5960519 |
| Otu_2 | 671 | 1 | 400 | 0.56 | 0.1422058 |
| **Otu_3** | **166** | **0.78** | **556** | **0.89** | **0.03748197*** |
| Otu_4 | 64 | 0.67 | 134 | 0.67 | 0.7187338 |
| Otu_5 | 105 | 1 | 65 | 0.67 | 0.6252367 |
| Otu_6 | 60 | 0.67 | 42 | 0.44 | 0.4585241 |
| Otu_7 | 12 | 0.22 | 62 | 0.67 | 0.08080837 |
| Otu_8 | 70 | 0.22 | 0 | 0 | 0.1456101 |
| Otu_9 | 0 | 0 | 67 | 0.11 | 0.3173105 |
| Otu_10 | 24 | 0.44 | 21 | 0.78 | 0.3606032 |
| Otu_11 | 44 | 0.22 | 0 | 0 | 0.1456101 |
| Otu_12 | 29 | 0.33 | 12 | 0.33 | 0.8727801 |
| Otu_13 | 10 | 0.56 | 22 | 0.44 | 0.9245757 |
| Otu_14 | 0 | 0 | 31 | 0.22 | 0.1456101 |
| **Otu_15** | **2** | **0.11** | **21** | **0.56** | **0.03528333*** |
| **Otu_16** | **21** | **0.56** | **0** | **0** | **0.01164329*** |
| Otu_17 | 0 | 0 | 21 | 0.11 | 0.3173105 |
| Otu_18 | 8 | 0.33 | 11 | 0.56 | 0.5257752 |
| **Otu_19** | **0** | **0** | **18** | **0.44** | **0.02845974*** |
| Otu_20 | 0 | 0 | 18 | 0.11 | 0.3173105 |
| Otu_21 | 0 | 0 | 17 | 0.22 | 0.1456101 |
| Otu_22 | 17 | 0.33 | 0 | 0 | 0.06619258 |
| Otu_23 | 15 | 0.67 | 2 | 0.22 | 0.03925033 |
| Otu_24 | 16 | 0.22 | 0 | 0 | 0.1449128 |
| Otu_25 | 1 | 0.11 | 14 | 0.22 | 0.4962425 |
| Otu_26 | 1 | 0.11 | 14 | 0.44 | 0.09314066 |
| Otu_27 | 0 | 0 | 13 | 0.11 | 0.3173105 |
| **Otu_28** | **0** | **0** | **12** | **0.67** | **0.00439749*** |
| Otu_29 | 5 | 0.11 | 6 | 0.56 | 0.100524 |
| Otu_30 | 0 | 0 | 10 | 0.11 | 0.3173105 |
| Otu_31 | 3 | 0.33 | 7 | 0.33 | 0.6347579 |
| Otu_32 | 0 | 0 | 10 | 0.11 | 0.3173105 |
| Otu_33 | 1 | 0.11 | 9 | 0.33 | 0.2249016 |
| **Otu_34** | **8** | **0.11** | **1** | **0.11** | **0.04409732*** |
| Otu_35 | 0 | 0 | 8 | 0.22 | 0.1456101 |
| Otu_36 | 2 | 0.22 | 6 | 0.11 | 0.633868 |
| Otu_37 | 5 | 0.22 | 2 | 0.11 | 0.4962425 |
| Otu_38 | 7 | 0.22 | 0 | 0 | 0.1456101 |
| Otu_39 | 0 | 0 | 7 | 0.11 | 0.3173105 |
| Otu_40 | 0 | 0 | 7 | 0.22 | 0.1456101 |
| Otu_41 | 4 | 0.22 | 3 | 0.11 | 0.5862137 |
| Otu_42 | 6 | 0.11 | 1 | 0.11 | 0.9355651 |
| Otu_43 | 0 | 0 | 6 | 0.22 | 0.1456101 |
| Otu_44 | 3 | 0.11 | 3 | 0.11 | 1 |
| Otu_45 | 3 | 0.22 | 3 | 0.33 | 0.7353167 |
| Otu_46 | 6 | 0.11 | 0 | 0 | 0.3173105 |
| Otu_47 | 6 | 0.22 | 0 | 0 | 0.1456101 |
| **Otu_48** | **6** | **0.44** | **0** | **0** | **0.02845974*** |
| Otu_49 | 6 | 0.22 | 0 | 0 | 0.1456101 |
| Otu_50 | 0 | 0 | 5 | 0.11 | 0.3173105 |
| Otu_51 | 0 | 0 | 5 | 0.11 | 0.3173105 |
| Otu_52 | 5 | 0.11 | 0 | 0 | 0.3173105 |
| Otu_53 | 5 | 0.22 | 0 | 0 | 0.1456101 |
| Otu_54 | 0 | 0 | 4 | 0.22 | 0.1456101 |
| Otu_55 | 0 | 0 | 4 | 0.33 | 0.06619258 |
| Otu_56 | 4 | 0.22 | 0 | 0 | 0.1456101 |
| Otu_57 | 3 | 0.22 | 1 | 0.11 | 0.4962425 |
| Otu_58 | 1 | 0.11 | 3 | 0.22 | 0.4962425 |
| Otu_59 | 4 | 0.33 | 0 | 0 | 0.06619258 |
| Otu_61 | 1 | 0.11 | 3 | 0.33 | 0.270485 |
| Otu_62 | 1 | 0.11 | 3 | 0.33 | 0.270485 |
| Otu_63 | 2 | 0.11 | 2 | 0.22 | 0.633868 |
| Otu_64 | 2 | 0.11 | 2 | 0.22 | 0.633868 |
| Otu_65 | 3 | 0.22 | 1 | 0.11 | 0.4962425 |
| Otu_66 | 2 | 0.22 | 2 | 0.22 | 1 |
| Otu_67 | 2 | 0.11 | 1 | 0.11 | 0.9355651 |
| Otu_68 | 1 | 0.11 | 2 | 0.22 | 0.5387949 |
| Otu_69 | 0 | 0 | 3 | 0.11 | 0.3173105 |
| Otu_70 | 1 | 0.11 | 2 | 0.22 | 0.5387949 |
| Otu_71 | 3 | 0.11 | 0 | 0 | 0.3173105 |
| Otu_73 | 0 | 0 | 3 | 0.33 | 0.06519642 |
| Otu_74 | 0 | 0 | 3 | 0.11 | 0.3173105 |
| Otu_75 | 2 | 0.11 | 1 | 0.11 | 0.9355651 |
| Otu_76 | 1 | 0.11 | 2 | 0.22 | 0.5387949 |
| Otu_78 | 0 | 0 | 3 | 0.33 | 0.06519642 |
| Otu_79 | 0 | 0 | 3 | 0.11 | 0.3173105 |
| Otu_80 | 2 | 0.11 | 1 | 0.11 | 0.9355651 |
| Otu_81 | 3 | 0.11 | 0 | 0 | 0.3173105 |
| Otu_82 | 0 | 0 | 3 | 0.11 | 0.3173105 |
| Otu_83 | 0 | 0 | 3 | 0.22 | 0.1456101 |
| Otu_84 | 3 | 0.22 | 0 | 0 | 0.1456101 |
| Otu_85 | 0 | 0 | 2 | 0.11 | 0.3173105 |
| Otu_86 | 0 | 0 | 2 | 0.11 | 0.3173105 |
| Otu_87 | 2 | 0.11 | 0 | 0 | 0.3173105 |
| Otu_88 | 1 | 0.11 | 1 | 0.11 | 1 |
| Otu_89 | 0 | 0 | 2 | 0.11 | 0.3173105 |
| Otu_91 | 2 | 0.22 | 0 | 0 | 0.1449128 |
| Otu_93 | 0 | 0 | 2 | 0.22 | 0.1449128 |
| Otu_94 | 0 | 0 | 2 | 0.11 | 0.3173105 |
| Otu_95 | 2 | 0.11 | 0 | 0 | 0.3173105 |
| Otu_97 | 1 | 0.11 | 1 | 0.11 | 1 |
| Otu_98 | 0 | 0 | 2 | 0.11 | 0.3173105 |
| Otu_100 | 1 | 0.11 | 1 | 0.11 | 1 |
| Otu_101 | 1 | 0.11 | 1 | 0.11 | 1 |
| Otu_102 | 0 | 0 | 2 | 0.11 | 0.3173105 |
| Otu_103 | 0 | 0 | 2 | 0.11 | 0.3173105 |
| Otu_104 | 1 | 0.11 | 1 | 0.11 | 1 |
| Otu_105 | 2 | 0.11 | 0 | 0 | 0.3173105 |
| Otu_106 | 0 | 0 | 2 | 0.22 | 0.1449128 |
| Otu_107 | 2 | 0.22 | 0 | 0 | 0.1449128 |
| Otu_108 | 0 | 0 | 2 | 0.11 | 0.3173105 |
| Otu_109 | 2 | 0.22 | 0 | 0 | 0.1449128 |
| Otu_110 | 2 | 0.22 | 0 | 0 | 0.1449128 |
| Otu_111 | 0 | 0 | 2 | 0.11 | 0.3173105 |
| Otu_112 | 1 | 0.11 | 1 | 0.11 | 1 |
| Otu_115 | 2 | 0.22 | 0 | 0 | 0.1449128 |
| Otu_116 | 0 | 0 | 2 | 0.11 | 0.3173105 |
| Otu_119 | 1 | 0.11 | 1 | 0.11 | 1 |
| Otu_120 | 2 | 0.11 | 0 | 0 | 0.3173105 |
| Otu_121 | 1 | 0.11 | 1 | 0.11 | 1 |
| Otu_123 | 0 | 0 | 2 | 0.22 | 0.1449128 |
| Otu_124 | 0 | 0 | 2 | 0.11 | 0.3173105 |
| Otu_125 | 0 | 0 | 2 | 0.22 | 0.1449128 |
| Otu_127 | 1 | 0.11 | 1 | 0.11 | 1 |
| Otu_129 | 0 | 0 | 2 | 0.11 | 0.3173105 |
| Otu_130 | 2 | 0.11 | 0 | 0 | 0.3173105 |
| Otu_132 | 0 | 0 | 2 | 0.11 | 0.3173105 |
| Otu_133 | 0 | 0 | 2 | 0.22 | 0.1449128 |
| Otu_134 | 2 | 0.22 | 0 | 0 | 0.1449128 |
| Otu_135 | 0 | 0 | 2 | 0.11 | 0.3173105 |
| Otu_136 | 2 | 0.11 | 0 | 0 | 0.3173105 |
| Otu_137 | 0 | 0 | 2 | 0.22 | 0.1449128 |
| Otu_138 | 0 | 0 | 2 | 0.11 | 0.3173105 |
| Otu_139 | 1 | 0.11 | 0 | 0 | 0.3173105 |
| Otu_140 | 0 | 0 | 1 | 0.11 | 0.3173105 |
| Otu_141 | 0 | 0 | 1 | 0.11 | 0.3173105 |
| Otu_144 | 1 | 0.11 | 0 | 0 | 0.3173105 |
| Otu_145 | 1 | 0.11 | 0 | 0 | 0.3173105 |
| Otu_146 | 1 | 0.11 | 0 | 0 | 0.3173105 |
| Otu_148 | 1 | 0.11 | 0 | 0 | 0.3173105 |
| Otu_152 | 0 | 0 | 1 | 0.11 | 0.3173105 |
| Otu_153 | 0 | 0 | 1 | 0.11 | 0.3173105 |
| Otu_154 | 0 | 0 | 1 | 0.11 | 0.3173105 |
| Otu_155 | 1 | 0.11 | 0 | 0 | 0.3173105 |
| Otu_158 | 1 | 0.11 | 0 | 0 | 0.3173105 |
| Otu_160 | 0 | 0 | 1 | 0.11 | 0.3173105 |
| Otu_163 | 1 | 0.11 | 0 | 0 | 0.3173105 |
| Otu_164 | 0 | 0 | 1 | 0.11 | 0.3173105 |
| Otu_165 | 1 | 0.11 | 0 | 0 | 0.3173105 |
| Otu_167 | 1 | 0.11 | 0 | 0 | 0.3173105 |
| Otu_169 | 0 | 0 | 1 | 0.11 | 0.3173105 |
| Otu_173 | 0 | 0 | 1 | 0.11 | 0.3173105 |
| Otu_174 | 0 | 0 | 1 | 0.11 | 0.3173105 |
| Otu_175 | 0 | 0 | 1 | 0.11 | 0.3173105 |
| Otu_177 | 0 | 0 | 1 | 0.11 | 0.3173105 |
| Otu_179 | 1 | 0.11 | 0 | 0 | 0.3173105 |
| Otu_180 | 0 | 0 | 1 | 0.11 | 0.3173105 |
| Otu_181 | 1 | 0.11 | 0 | 0 | 0.3173105 |
| Otu_182 | 0 | 0 | 1 | 0.11 | 0.3173105 |
| Otu_183 | 0 | 0 | 1 | 0.11 | 0.3173105 |
| Otu_184 | 0 | 0 | 1 | 0.11 | 0.3173105 |
| Otu_189 | 0 | 0 | 1 | 0.11 | 0.3173105 |
| Otu_191 | 0 | 0 | 1 | 0.11 | 0.3173105 |
| Otu_192 | 0 | 0 | 1 | 0.11 | 0.3173105 |
| Otu_200 | 0 | 0 | 1 | 0.11 | 0.3173105 |
| Otu_201 | 0 | 0 | 1 | 0.11 | 0.3173105 |
| Otu_203 | 0 | 0 | 1 | 0.11 | 0.3173105 |
| Otu_204 | 0 | 0 | 1 | 0.11 | 0.3173105 |
| Otu_205 | 1 | 0.11 | 0 | 0 | 0.3173105 |
| Otu_207 | 0 | 0 | 1 | 0.11 | 0.3173105 |
| Otu_208 | 0 | 0 | 1 | 0.11 | 0.3173105 |
| Otu_212 | 1 | 0.11 | 0 | 0 | 0.3173105 |
| Otu_213 | 0 | 0 | 1 | 0.11 | 0.3173105 |
| Otu_214 | 1 | 0.11 | 0 | 0 | 0.3173105 |
| Otu_215 | 0 | 0 | 1 | 0.11 | 0.3173105 |
| Otu_218 | 1 | 0.11 | 0 | 0 | 0.3173105 |
| Otu_224 | 1 | 0.11 | 0 | 0 | 0.3173105 |
| Otu_226 | 1 | 0.11 | 0 | 0 | 0.3173105 |
| Otu_228 | 1 | 0.11 | 0 | 0 | 0.3173105 |
| Otu_234 | 1 | 0.11 | 0 | 0 | 0.3173105 |
| Otu_235 | 0 | 0 | 1 | 0.11 | 0.3173105 |
| Otu_236 | 0 | 0 | 1 | 0.11 | 0.3173105 |
| Otu_237 | 1 | 0.11 | 0 | 0 | 0.3173105 |
| Otu_238 | 0 | 0 | 1 | 0.11 | 0.3173105 |
| Otu_239 | 1 | 0.11 | 0 | 0 | 0.3173105 |
| Otu_240 | 1 | 0.11 | 0 | 0 | 0.3173105 |
| Otu_241 | 0 | 0 | 1 | 0.11 | 0.3173105 |
| Otu_247 | 1 | 0.11 | 0 | 0 | 0.3173105 |
| Otu_248 | 0 | 0 | 1 | 0.11 | 0.3173105 |
| Otu_251 | 1 | 0.11 | 0 | 0 | 0.3173105 |
| Otu_254 | 0 | 0 | 1 | 0.11 | 0.3173105 |
| Otu_264 | 1 | 0.11 | 0 | 0 | 0.3173105 |
| Otu_265 | 1 | 0.11 | 0 | 0 | 0.3173105 |
| Otu_266 | 1 | 0.11 | 0 | 0 | 0.3173105 |
| Otu_267 | 1 | 0.11 | 0 | 0 | 0.3173105 |
| Otu_268 | 1 | 0.11 | 0 | 0 | 0.3173105 |
| Otu_269 | 1 | 0.11 | 0 | 0 | 0.3173105 |
| Otu_272 | 0 | 0 | 1 | 0.11 | 0.3173105 |
| Otu_273 | 1 | 0.11 | 0 | 0 | 0.3173105 |
| Otu_274 | 1 | 0.11 | 0 | 0 | 0.3173105 |
| Otu_277 | 0 | 0 | 1 | 0.11 | 0.3173105 |
| Otu_279 | 0 | 0 | 1 | 0.11 | 0.3173105 |
| Otu_280 | 1 | 0.11 | 0 | 0 | 0.3173105 |
| Otu_281 | 0 | 0 | 1 | 0.11 | 0.3173105 |
| Otu_282 | 1 | 0.11 | 0 | 0 | 0.3173105 |
| Otu_283 | 1 | 0.11 | 0 | 0 | 0.3173105 |
| Otu_285 | 1 | 0.11 | 0 | 0 | 0.3173105 |
| Otu_286 | 1 | 0.11 | 0 | 0 | 0.3173105 |
| Otu_287 | 1 | 0.11 | 0 | 0 | 0.3173105 |
| Otu_289 | 1 | 0.11 | 0 | 0 | 0.3173105 |
| Otu_294 | 0 | 0 | 1 | 0.11 | 0.3173105 |
| Otu_296 | 1 | 0.11 | 0 | 0 | 0.3173105 |
| Otu_299 | 0 | 0 | 1 | 0.11 | 0.3173105 |
| Otu_302 | 1 | 0.11 | 0 | 0 | 0.3173105 |
| Otu_307 | 1 | 0.11 | 0 | 0 | 0.3173105 |
| Otu_308 | 0 | 0 | 1 | 0.11 | 0.3173105 |
| Otu_310 | 0 | 0 | 1 | 0.11 | 0.3173105 |
| Otu_313 | 1 | 0.11 | 0 | 0 | 0.3173105 |
| Otu_314 | 1 | 0.11 | 0 | 0 | 0.3173105 |
| Otu_316 | 0 | 0 | 1 | 0.11 | 0.3173105 |
| Otu_317 | 1 | 0.11 | 0 | 0 | 0.3173105 |
| Otu_318 | 1 | 0.11 | 0 | 0 | 0.3173105 |
| Otu_319 | 0 | 0 | 1 | 0.11 | 0.3173105 |
| Otu_320 | 0 | 0 | 1 | 0.11 | 0.3173105 |
| Otu_322 | 0 | 0 | 1 | 0.11 | 0.3173105 |

^2^ statistics were performed using Kruskal-Wallis' test. ‘*’ P < 0.05; ‘ns’ P > 0.05. In bold OTUs presenting a significant difference between the two sites.
