# Supplementary material for: Molecular Characterization of Arbuscular Mycorrhizal Fungi in an Agroforestry System Reveals the Predominance of Funneliformis spp. Associated with Colocasia esculenta and Pterocarpus officinalis Adult Trees and Seedlings
Source: Front Microbiol. 2017 Jul 28;8:1426. doi: 10.3389/fmicb.2017.01426 (PMC5532380; doi:10.3389/fmicb.2017.01426)
Supplement: Supplementary file 7 [file Table_5.DOCX]

**Table S5.** AM fungal OTUs preferentially associated with a locality (Grande Ravine or Belle Plaine)

| Locality | Indicator taxa  Taxonomy (OTU label) | Frequency ^1^  (BP / GR) | r.g ^2^ | P*-value ^3^* |
| --- | --- | --- | --- | --- |
| Belle Plaine | *Gigaspora* (OTU_28) | 0.7 / 0.0 | 0.577 | 0.0091^*^ |
|  | *Gigaspora* (OTU_15) | 0.6 / 0.1 | 0.508 | 0.0469^*^ |
|  | *Acaulospora* (OTU_3) | 0.9 / 0.8 | 0.432 | 0.0425^*^ |
| Grande Ravine | *Rhizophagus* (OTU_16) | 0.0 / 0.6 | 0.321 | 0.0274 |

^1^ Frequency indicates the frequency of an OTU in samples of a given locality. BP, Belle Plaine; GR, Grande Ravine.

^2^ The corrected Pearson’s phi coefficient of association (“r.g”) was used as model to determine indicator OTUs.

^3^ ‘*’ *P* < 0.05; ‘ns’ *P* > 0.05
