# Supplementary material for: Molecular Characterization of Arbuscular Mycorrhizal Fungi in an Agroforestry System Reveals the Predominance of Funneliformis spp. Associated with Colocasia esculenta and Pterocarpus officinalis Adult Trees and Seedlings
Source: Front Microbiol. 2017 Jul 28;8:1426. doi: 10.3389/fmicb.2017.01426 (PMC5532380; doi:10.3389/fmicb.2017.01426)
Supplement: Supplementary file 8 [file Table_6.DOCX]

**Table S6**. Single and pair of AM fungal OTUs associated with a plant type in *Pterocarpus*-taro in Guadeloupean agroforestry systems.

| Locality | Plant | Indicator taxa  Taxonomy (OTU) | Frequency ^1^  (P.a / P.s / T) | A ^1^  (specificity) | B  (sensibility) | IndVal.g | P-value ^3^ |
| --- | --- | --- | --- | --- | --- | --- | --- |
| Belle Plaine | T. | Gigaspora (33) | 0.0 / 0.0 / 1.0 | 1.00 | 1.00 | 1.00 | 0.027^*^ |
|  |  | Funneliformis (1) + Gigaspora (33) |  | 1.00 | 1.00 | 1.00 | 0.027^*^ |
|  |  | Gigaspora (7) + Gigaspora (33) |  | 1.00 | 1.00 | 1.00 | 0.027^*^ |
|  |  | Archaeospora (10) + Gigaspora (33) |  | 1.00 | 1.00 | 1.00 | 0.027^*^ |
|  |  | Gigaspora (15) + Gigaspora (26) |  | 1.00 | 1.00 | 1.00 | 0.027^*^ |
|  |  | Gigaspora (15) + Gigaspora (33) |  | 1.00 | 1.00 | 1.00 | 0.027^*^ |
|  |  | Gigaspora (26) + Gigaspora (33) |  | 1.00 | 1.00 | 1.00 | 0.027^*^ |
|  | P.a | Acaulospora (13) + Gigaspora (28) |  | 1.00 | 1.00 | 1.00 | 0.036^*^ |
| Grande Ravine | T. | Gigaspora (22) | 0.0 / 0.0 / 1.0 | 1.00 | 1.00 | 1.00 | 0.035^*^ |
|  |  | Funneliformis (1) + Gigaspora (22) |  | 1.00 | 1.00 | 1.00 | 0.035^*^ |
|  |  | Incertae sedis Glomus (2) + Gigaspora (22) |  | 1.00 | 1.00 | 1.00 | 0.035^*^ |
|  |  | Incertae sedis Glomus (4) + Gigaspora (22) |  | 1.00 | 1.00 | 1.00 | 0.035^*^ |
|  |  | Funneliformis (5) + Gigaspora (22) |  | 1.00 | 1.00 | 1.00 | 0.035^*^ |
|  |  | Incertae sedis Glomus (6) + Gigaspora (22) |  | 1.00 | 1.00 | 1.00 | 0.035^*^ |
|  |  | Gigaspora (22) + Rhizophagus (23) |  | 1.00 | 1.00 | 1.00 | 0.035^*^ |
|  |  | Gigaspora (22) + Funneliformis (48) |  | 1.00 | 1.00 | 1.00 | 0.035^*^ |
|  | P.a | Geosiphon (18) | 0.0 / 0.0 / 1.0 | 1.00 | 1.00 | 1.00 | 0.032^*^ |
|  |  | Funneliformis (1) + Geosiphon (18) |  | 1.00 | 1.00 | 1.00 | 0.032^*^ |
|  |  | Incertae sedis Glomus (2) + Geosiphon (18) |  | 1.00 | 1.00 | 1.00 | 0.032^*^ |
|  |  | Acaulospora (3) + Archaeospora (10) |  | 1.00 | 1.00 | 1.00 | 0.032^*^ |
|  |  | Acaulospora (3) + Geosiphon (18) |  | 1.00 | 1.00 | 1.00 | 0.032^*^ |
|  |  | Funneliformis (5) + Geosiphon (18) |  | 1.00 | 1.00 | 1.00 | 0.032^*^ |
|  |  | Archaeospora (10) + Geosiphon (18) |  | 1.00 | 1.00 | 1.00 | 0.032^*^ |
|  |  | Archaeospora (10) | 1.0 / 0.0 / 0.3 | 0.96 | 1.00 | 0.98 | 0.032^*^ |
|  |  | Funneliformis (1) + Archaeospora (10) |  | 0.96 | 1.00 | 0.98 | 0.032^*^ |
|  |  | Incertae sedis Glomus (2) + Archaeospora (10) |  | 0.96 | 1.00 | 0.98 | 0.032^*^ |
|  | T. + P.s | Incertae sedis Glomus (4) | 0.0 / 1.0 / 1.0 | 1.00 | 1.00 | 1.00 | 0.032^*^ |
|  |  | Rhizophagus (23) | 0.0 / 1.0 / 1.0 | 1.00 | 1.00 | 1.00 | 0.032^*^ |
|  |  | Funneliformis (1) + Incertae sedis Glomus (4) |  | 1.00 | 1.00 | 1.00 | 0.032^*^ |
|  |  | Funneliformis (1) + Rhizophagus (23) |  | 1.00 | 1.00 | 1.00 | 0.032^*^ |
|  |  | Incertae sedis Glomus (2) +  Incertae sedis Glomus (4) |  | 1.00 | 1.00 | 1.00 | 0.032^*^ |
|  |  | Incertae sedis Glomus (2) + Rhizophagus (23) |  | 1.00 | 1.00 | 1.00 | 0.032^*^ |
|  |  | Incertae sedis Glomus (4) + Funneliformis (5) |  | 1.00 | 1.00 | 1.00 | 0.032^*^ |
|  |  | Incertae sedis Glomus (4) + Rhizophagus (23) |  | 1.00 | 1.00 | 1.00 | 0.032^*^ |
|  |  | Funneliformis (5) + Rhizophagus (23) |  | 1.00 | 1.00 | 1.00 | 0.032^*^ |

^1^ Frequency indicates the presence of an OTU in samples of a given plant type. P.a, *Pterocarpus* adult tree; P.s, *Pterocarpus* seedling; T., taro

^3^ The corrected Indval coefficient of association (“IndVal.g”) was used as model to determine indicator OTUs.

^3^ ‘*’ *P* < 0.05; ‘ns’ *P* > 0.05
