# Supplementary material for: Molecular Characterization of Arbuscular Mycorrhizal Fungi in an Agroforestry System Reveals the Predominance of Funneliformis spp. Associated with Colocasia esculenta and Pterocarpus officinalis Adult Trees and Seedlings
Source: Front Microbiol. 2017 Jul 28;8:1426. doi: 10.3389/fmicb.2017.01426 (PMC5532380; doi:10.3389/fmicb.2017.01426)
Supplement: Supplementary file 9 [file Data_Sheet_1.DOCX]

**MOLECULAR CHARACTERIZATION OF ARBUSCULAR MYCORRHIZAL FUNGI IN AN AGROFORESTRY SYSTEM REVEALS THE PREDOMINANCE OF *FUNNELIFORMIS* SPP. ASSOCIATED WITH *COLOCASIA ESCULENTA* AND *PTEROCARPUS OFFICINALIS* ADULT TREES AND SEEDLINGS**

Alexandre Geoffroy ^1, 2^*, Hervé Sanguin ^2^, Antoine Galiana ^2^ and Amadou Bâ ^1^

^1^ Université des Antilles, UMR LSTM, Laboratoire de Biologie et Physiologie Végétales, UFR Sciences, BP. 592, 97159 Pointe-à-Pitre, Guadeloupe, France.

^2^ CIRAD, UMR LSTM, Montpellier, France

* Corresponding author:

Dr Alexandre Geoffroy

E-mail address: [alex21.geoffroy@gmail.com](mailto:alex21.geoffroy@gmail.com)

Number of words: 4042

Number of figures: 3

Running title: *Funneliformis* predominates in *Pterocarpus-*taro agroforestry systems

**Abstract**

*Pterocarpus officinalis* (Jacq.) is a leguminous forestry tree species endemic to Caribbean swamp forests. In Guadeloupe, smallholder farmers traditionally cultivate flooded taro (*Colocasia esculenta*) cultures under the canopy of *P. officinalis* stands. The role of arbuscular mycorrhizal (AM) fungi in the sustainability of this traditional agroforestry system has been suggested but the composition and distribution of AM fungi colonizing the leguminous tree and/or taro are poorly characterized. An in-depth characterization of root-associated AM fungal communities from *P. officinalis* adult trees and seedlings and taro cultures, sampled in two localities of Guadeloupe, was performed by pyrosequencing (GS FLX+) of partial 18S rRNA gene. The AM fungal community was composed of 215 operational taxonomic units (OTUs), belonging to eight fungal families dominated by Glomeraceae, Acaulosporaceae and Gigasporaceae. Results revealed a low AM fungal community membership between *P. officinalis* and *C. esculenta*. However, certain AM fungal community taxa (10% of total community) overlapped between *P. officinalis* and *C. esculenta*, notably predominant *Funneliformis* OTUs. These findings provide new perspectives in deciphering the significance of *Funneliformis* in nutrient exchange between *P. officinalis* and *C. esculenta* by forming a potential mycorrhizal network.

**Keywords**: Arbuscular mycorrhizal community, *Colocasia esculenta*, *Funneliformis*, Guadeloupe, *Pterocarpus officinalis*, Pyrosequencing, Tropical agroforestry

**Introduction**

*Pterocarpus officinalis* L. is one of the dominant wetland tree species of the seasonally flooded swamp forests in the Caribbean and the Guiana regions (Eusse and Aide 1999, Bâ and Rivera-Ocasio 2015). It covers large areas of the coastal floodplain as individual trees and small patches adjacent to mangroves, and along rivers and in mountains (Eusse and Aide 1999). In the Caribbean, this unique *P. officinalis* swamp forest provides a habitat for many species of plants and animals and reduces soil erosion along the margins and riverbanks in coastal and mountain areas (Saur and Imbert 2003, Bâ and Rivera-Ocasio 2015). Despite its ecological interest, most of the populations of *P. officinalis* in the Caribbean islands are restricted to a small area due to wetland drainage and urban development (Bâ and Rivera-Ocasio 2015). Furthermore, the low genetic diversity found within and between populations of *P. officinalis* is exacerbated by a strong inbreeding depression (Muller et al. 2009). As a consequence, management and conservation measures must be implemented to preserve the remaining *P. officinalis* populations.

In some Caribbean islands, a dominant management strategy for *P. officinalis* conservation is to plant agricultural crops under these stands. The Guadeloupean smallholder farmers notably conduct taro (*Colocasia esculenta* L. Schott) monocultures under the *P. officinalis* stands in freshwater flooding swamp forests because of higher crop yields compared to other agricultural practices (Saur and Imbert 2003). No fertilizer or pesticide are used, and the *P. officinalis* trees are not impacted (no cutting), preserving the tree genetic diversity, and the ecosystem processes (e.g. nutrient cycling, local biodiversity) (Rivera-Ocasio et al. 2006, Bâ and Rivera-Ocasio 2015). The ecological mechanisms sustaining the functioning of this traditional agroforestry system remains poorly investigated. Indeed heavy leaching of soils brought by seasonal flooding contributes to the shortage of available P and N and should be detrimental for taro monocultures (Bâ and Rivera-Ocasio 2015). *Pterocarpus* stands might be beneficial to understory taro cultures by (i) maintaining humidity and temperature at certain levels to prevent water stress (Sanou et al. 2012), and (ii) improving the N input on soil and non-legume plants through biological nitrogen fixation process (Koponen et al. 2003, Saur and Imbert 2003). The transfer of N from legume to the non-legume can occurred through root exudation, root and nodule decomposition and mineralization, as well as mediated by plant-associated AM fungi (Kaiser et al. 2015). Both *P. officinalis* and *C. esculenta* establish a symbiosis with AM fungi (Saint-Etienne et al. 2006, Wang and Qiu 2006, Fougnies et al. 2007), notably improving plant phosphorus (P) nutrition (Smith and Read 2008), but also potentially plant N nutrition (Hodge et al 2001, Walder et al. 2012). In addition, *P. officinalis* requires P from AM fungi not only for their nutrition but also for efficient nodule formation and nitrogen fixation (Fougnies et al. 2007, Le Roux et al. 2014). Whereas, the diversity of nitrogen-fixing bacteria associated with *P. officinalis* in Caribbean swamp forests has been described (Le Roux et al. 2014), AM fungal diversity has been poorly investigated.

AM fungi are the most common and widespread symbiosis involving 86% of land plants including many important crops (Davison et al. 2012). The extraradical phase of AM fungi acts as an extension of the root system for the uptake of nutrients in exchange for plant-synthesized carbon. AM fungi are assumed not host specific symbiosis but a given AM taxa could have different effects depending on plant species (Thioye et al. 2017). The relatively low host specificity of AM fungi, increases the possibility that extraradical fungal hyphae links multiple plant species to form common mycorrhizal networks (CMNs) in a plant community (Cheng and Baumgartner 2004, Ingleby et al. 2007). Mycorrhizal networks are known to drive nutrient transfers (mainly C and N) between adult trees and seedlings for one plant species, and among different plants species (e.g. legume and non-legume) (Cheng and Baumgartner 2004, He et al. 2004, Selosse et al. 2006, Wahbi et al. 2016). However, mycorrhizal networks have been mainly assessed at the fungal strain level in controlled conditions (Walder et al. 2012) and rarely at the community level (Montesinos-Navarro et al. 2012). Thanks to the development of high-throughput sequencing approaches such as pyrosequencing, the complexity of AM fungal community among plant roots can be deeply assessed (Hart et al. 2015), and a wide range of ecological studies based on the diversity of AM fungal taxonomic markers such as the SSU rRNA gene has been performed (Öpik et al. 2006, 2014).

The current study aims the molecular characterization of AM fungal community composition and distribution between *P. officinalis* and *C. esculenta* crops in a traditional Guadeloupean agroforestry system (swamp forests) in order to evaluate if the sustainability of the system might be explained by a high similarity or a high dissimilarity of AM fungal community between *Pterocarpus* and taro. In addition, the comparison of AM fungal community of *P. officinalis* adult trees and seedlings with the ones of taro were investigated because of the potential importance of seedlings in the sustainability of the agroecosystems since they are conserved inside the taro cultures. Consequently, two main questions were assessed, (i) What are the predominant mycorrhizal taxa in the traditional agroforestry system, and (ii) What is the degree of similarity of AM fungal members among *P. officinalis* adult trees, seedlings and taro?

**Materials and methods**

**Study sites and sampling**

We conducted the sampling during 2012 in two representative *Pterocarpus* swamp forests located in the Grande-Terre island of Guadeloupe: Grande Ravine (GR) (16°13’N, 61°28’W) and Belle Plaine (BP) (16°17’N, 61°31’W) (Figure S1). The *P. officinalis* stands, which comprise 45 and 52 adult trees for GR and BP sites respectively, are fairly forested and contain dense populations of understory regenerating seedlings. The GR forest site (approximately 0.3 ha) is located along the GR river and taro plants (*Colocasia* *esculenta*) were cultivated by smallholders farmers under adult trees and between naturally regenerating seedlings. Some individuals of understory plant species such as *Ficus* sp.1, *Commelina* sp.1 and *Mimosa pudica* were naturally associated with *P. officinalis*. The BP forest site (approximately 0.4-ha), is located around the bay of the Grand Cul-de-sac Marin, in the near mangrove area and taro plants were also cultivated between *Pterocarpus* trees and regenerating seedlings. Understory species like *Musa* sp. and *Ficus* sp. are represented by a few individuals and are widely spaced from one another. In each forest site, soil cores (200 g of fresh soil) were randomly collected near three adult trees (more than 25 m high), three seedlings (1 < height < 2 m) and three taro plants. Overall, 18 soil cores were stored at 4°C before being processed. Roots were separated from soil, gently washed with tap water and dried with Silica-gel until molecular analyses. Soil physico-chemical parameters were measured at the Celesta-lab (Mauguio, France) (Table S1).

**Molecular analyses**

For each root sample, the three replicates were pooled and subjected to liquid nitrogen grinding for homogenization. Total DNA was extracted from a sub-sample (100 mg of dried root) using a FastPrep-24 homogenizer (MP biomedicals Europe, Illkirch, France) and the FastDNA® SPIN kit (MP biomedicals Europe) according to manufacturer’s instructions. The quality of DNA extracts was improved by adding 20-30 mg Polyvinylpolypyrrolidon (PVPP) during the first step of DNA extraction. Two DNA extractions were done per root sample.

*Glomeromycota* (AM fungi) sequences were amplified using the nuclear 18S rRNA gene primers NS31 (5′- (10-bp MID) TTGGAGGGCAAGTCTGGTGCC -3′) (Simon et al. 1992) and AML2 (5′- (10-bp MID) GAACCCAAACACTTTGGTTTCC -3′) (Lee et al. 2008). MIDs (multiplex identifier) were designed by Eurofins Genomics (Eurofins Genomics GmbH, www.eurofinsgenomics.eu). PCR conditions were performed according to Sanguin et al (2016). Two PCR products per root sample were pooled before purification using illustra GFX PCR DNA and Gel Band Purification Kit (GE Healthcare Life Sciences, Velizy-Villacoublay, France) following manufacturer’s guidelines. Overall 18 PCR products were subjected to bi-directional 454-sequencing (1/4th plate Roche GS FLX+ run using the GS FLX Titanium sequencing kit XL+) by Eurofins Genomics.

**Data processing and taxonomic assignment of AM fungal sequences**

454-sequencing data were analyzed using Mothur software according the standard operating procedure (http://www.mothur.org/wiki/454_SOP) proposed (Schloss et al. 2011), except for the quality cutoffs, for which it has been set up at Q30 (*trim.seqs* command). All sequence reads were then depleted of barcodes and primers (final length 230 bp), and sequences < 100 bp or with ambiguous base calls or with homopolymer runs exceeding 8 bp were also removed. A pre-clustering step (Huse et al. 2010) was also performed to remove sequences still likely due to pyrosequencing errors. Chimeric sequences were checked by using UCHIME (Edgar et al. 2011) and removed. Finally, sequences were identified using the Glomeromycota-based alignment database (Krüger et al. 2012) and sequence similarity ≥60% at the family level.

Clustering of sequences in operational taxonomic unit (OTUs) was performed using *dist.seqs* and cluster commands in Mothur. Then, the number of sequences from each sample was normalized with *sub.sample* command. This sub-sampling step allows reducing the number of spurious OTUs and is crucial to obtain robust estimation of alpha and beta diversity (Gihring et al. 2012). Finally, OTUs were defined at 97% similarity level for taxonomic affiliation.

**Statistical analyses**

Diversity (Shannon, inverse Simpson [1/D]), richness (number of OTUs, Chao1) and evenness (Pielou) indexes were estimated using R version 3.3.2 (R Core Team 2016) and the R package vegan (Oksanen et al. 2016). The sequencing effort was evaluated using the coverage calculator and Boneh estimator (Boneh et al. 1998) implemented in Mothur. We assessed the effects of forest site, plant species, *Pterocarpus* age categories and their interactions on the AM fungal community composition by non-parametric permutational multivariate analysis of variance (PERMANOVA) (Anderson 2001) implemented in the *perm.anova*() function from the R package RVAideMemoire (Hervé 2016). The differences in AM fungal community structure among forest sites and plant species were assessed using PERMANOVA in *adonis*() function (McArdle and Anderson 2001), both from the R package vegan. The AM fungal community structure was based on the Bray-Curtis dissimilarity index as defined in *vegdist*() function from the R package vegan. Multivariate dispersion was estimated using the *betadisper*() and *permutest*() functions (999 permutations; alpha = 0.05) from the R package vegan because it can affect PERMANOVA results. Differences in the relative abundances of AM fungal OTUs among forest sites or plant species were estimated using Kruskal-Wallis’ test implemented in *kruskal.test*() function from the R package stats. The frequency of AM fungal OTUs was determined using the *strassoc*() function from the R package indicspecies (De Cáceres and Legendre 2009).

The determination of AM fungal OTUs preferentially associated with a given forest site was performed using the corrected Pearson’s phi coefficient of association (“r.g”) implemented in the *multipatt*() function (De Cáceres et al. 2010) from the R package indicspecies. AM fungal OTUs preferentially associated with a given plant species was assessed using the corrected indicator value index (“IndVal.g”species) from the R package indicspecies. A procedure based on determination of species (i.e. OTU) and group (i.e. plant type) combinations was applied using successively *combinespecies*() and *multipatt*() functions. This procedure was demonstrated to bear more ecological informations and to determine more robust predictive indicator value than by considering species or group independently (De Cáceres et al. 2012). Two different probabilities were calculated, *i.e.* A (specificity), representing the probability of a sample to be defined by a group (i.e. plant type), given that the species or the species combinations have been detected, and B (sensitivity) representing the probability of finding the species or the species combinations in different samples characterized by a given group (i.e. plant type). Only AM fungal OTUs present in 2 samples among 3 groups defined (i.e. plant type) were subjected to analysis. We considered as valid indicators the OTUs showing both A (specificity) and B (sensitivity) superior to 0.8 and 0.6 respectively, as recommended in Suz et al. (2014).

The AM fungal community membership among *C. esculenta* and *P. officinalis* adult trees and seedlings was assessed using venn diagram analysis with the R package VennDiagram (Chen 2016), and the relative abundance of AM fungal taxa shared among plants was characterized using bipartite network analysis with the *plotweb*() function from the R package bipartite (Dorman et al. 2008).

**Results**

**AM fungal community composition among forest sites**

The global 454-pyrosequencing data were composed of 210,676 reads, and 155,544 reads (74 %) passed the quality control steps. The average read length was 230 bp. After trimming, pre-clustering and chimera detection steps, 70,949 sequences were classified using a Glomeromycota-based alignment database. A total of 31,803 non-Glomeromycota sequences were removed from the dataset (70,949), as well as singletons (1515 sequences) that are mostly considered as artefacts and can lead to overestimations of AM fungal diversity. The AM fungal sequences (37,631 reads) were assigned to a total of 215 OTUs based on a sequence similarity threshold ≥ 97%. The sequence number between samples was rarefied to 440 sequences per sample (threshold based on the sample with the lower number of sequences) to improve statistical robustness. A high diversity coverage (94-98%) was reached for all samples, with less than eight potential OTUs that were not retrieved (Boneh estimation, Table S2). The Boneh estimation showed that the sequencing depth was sufficient to estimate and compare the microbial diversity of the samples (Table S2).

The taxonomy assignment of OTUs (Table S3) revealed a main affiliation to Glomeraceae (83.5% of sequences), but also to Acaulosporaceae (9.7%), Gigasporaceae (5.3%), Archaesporaceae (0.9%), Geosiphonaceae (0.3%), Diversisporaceae (0.1%), Paraglomeraceae (0.05%), and Pacisporaceae (0.03%). In Glomeraceae, *Funneliformis* represents the most abundant genus (62% of sequences), followed by fungi of uncertain position in *Glomus* sensus lato (20 %), *Rhizophagus* (2 %) and *Sclerocystis* / *Glomus* (< 1%) (Table S3). The low evenness estimated for all AM fungal communities (< 0.3) revealed the strong predominance of few OTUs and numerous rare OTUs (Table S2). Three OTUs, *i.e.* OTU1 and OTU2, belonging to Glomeraceae and OTU3 to Acaulosporaceae families, represented more than 80% of sequences (Table S3). The most predominant OTU (OTU1, 58% of sequences) was affiliated to *Funneliformis*.

Richness, diversity and evenness were calculated for the different AM fungal communities. No significant difference was observed at both forest sites (Table S2). AM fungal community structure analysis, based on Bray-curtis index, also showed no significant difference between both forest sites (Table 2). However, four OTUs affiliated to *Acaulospora, Gigaspora*, and *Incertae sedis Glomus* showed significantly higher abundance in BP, and three OTUs affiliated to *Rhizophagus* and *Funneliformis* in GR (Figure 1, for all comparisons see Table S4). Among the 215 AM fungal OTUs, only four were determined as preferentially associated at a given forest site (Table S5), among which two of them, *i.e.* *Gigaspora* (OTU28) and *Rhizophagus* (OTU16) were exclusively found in BP and GR sites, respectively.

**AM fungal community composition among plant types**

AM fungal community richness (for the two indices Chao1 and OTUs number) was significantly different between taro and *Pterocarpus* (*P*=0.033; Table S2) whereas only a locality-dependent plant type effect was observed on AM fungal community structures (*P* = 0.011; Table 1). The analysis of AM fungal community structure for each forest site confirmed the plant type effect for the GR forest site (*P* = 0.036) (Table 1). The NMDS analysis (Figure S2), taking into account all OTUs, highlighted the dissimilarity among AM fungal communities of *Pterocarpus* adult trees compared to both taro and *Pterocarpus* seedlings in the GR site. Out of a total of 7750 and 9730 AM fungal OTUs and OTU pair combinations for the BP and GR sites, 8 and 27 AM fungal indicators were determined, respectively (Table 2 and table S6), confirming the significant and preferential association of *Gigaspora* (OTU22 and OTU33) with taro. In the GR forest site, six AM fungal OTUs (OTU1, OTU2, OTU4, OTU5, OTU6 and OTU48) belonging to the Glomeraceae were significantly associated with both taro and *Pterocarpus* seedlings, and two AM fungal OTUs (OTU18 and OTU3) with *Pterocarpus* adult trees.

Venn diagram analysis of OTU-based AM fungal community revealed a low membership characterized by a high number of OTUs specific to one plant and few overlapping OTUs among taro and the two age categories of *Pterocarpus*, i.e. 10% and 8% of common OTUs in BP and GR sites, respectively (Figure 2A). Taro showed the highest number of specific OTUs (> 35%) compared to *Pterocarpus* (< 25%) in both forest sites. Bipartite network analysis showed that the overlapping AM fungi between plants species was mainly composed of *Funneliformis* OTUs, with 53 % and 61 % of sequences in BP and GR forest sites, respectively (Figure 2B). Rare OTUs mainly constituted the plant-specific OTUs, which fit with the low number of indicator taxa associated with the different types of plants.

**Discussion**

AM fungi might play a major role in the functioning of the traditional Guadeloupean agroforestry system associating *P. officinalis* trees, their naturally regenerating seedlings, and an understory taro monoculture, and the degree AM fungal community similarity among plants has been hypothesized as one of the main factors. Significant differences in AM fungal community richness and structure were observed among the different plant types, but not in terms of diversity. Some AM fungal OTUs were preferentially associated with a given plant type, but a highly predominant AM fungal OTU affiliated to *Funneliformis* was detected among all plants.

**Characteristics of AM fungal community**

A relatively high AM fungal richness ( > 120 OTUs) were observed compared to other AM fungal surveys using high-throughput methods from a wide range of in tropical forest ecosystems (22 to 207 OTUs; De Beenhouwer et al. 2015, Holste et al. 2016, Rodrίguez-Echeverrίa et al. 2016). As shown by Bainard et al. (2011), tree-based cropping systems, combining different tree species (white ash, hybrid poplar and Norway spruce) with annual crops (corn, soybean and winter wheat), can present a highly diverse AM fungal community. However, the robustness of comparisons between different tropical agro-ecosystems remains questionable due to the scarcity of studies in tropical regions. Furthermore, OTU-based fungal richness is highly dependent on the bioinformatics treatment applied (mainly sequence quality filtering and clustering methods) (Balint et al. 2016); the biological material analyzed (roots, spores and extraradical mycelium) (Varela-Cervero et al. 2015) or the methodology used (spore identification, PCR-cloning, pyrosequencing) (Davison et al. 2015). The predominance of Glomeraceae, which is the most widespread family in natural and managed ecosystems (Oehl et al. 2010, Brearley et al. 2016) and Acaulosporaceae was in agreement with several surveys carried out in tropical environments (Leal et al. 2013, De Beenhouwer et al. 2015, Holste et al. 2016). Only 4% of all detected AM fungal OTUs were poorly affiliated to a reference taxa in databases, which contrasted with previous data in dry afromontane forests (Wubet et al. 2003) and dry tropical regions (Rodrίguez-Echeverrίa et al. 2016), where up to 18% and 15% of OTUs were considered as new taxa, respectively.

Characteristics of AM fungal community (diversity, richness, structure) were similar between the two forest sites (separated of 15 km), suggesting a global AM fungal community pattern for this agroforestry system. However, some AM fungal were associated to a given forest site, notably for BP site. The soil characteristics and soil hypoxia are known as major drivers of AM fungal community (Helgason and Fitter 2009, Oehl et al. 2010, Aguacil et al. 2016) and differences observed between the both forest sites in soil nutrient contents (N, Ca and Na; Table S1) and flooding duration might have favored specific taxa. The low number of indicator species observed in our work was consistent with the study by Moora et al. (2014), which showed that forest plantations or cultivated lands have very few AM indicator species compared to primary forests or permanent grasslands.

**Degree of AM fungal community similarity between tree and culture**

The analysis of AM fungal community composition and structure demonstrated a site-dependent host plant effect due to low abundant OTUs notably belonging to *Gigaspora* for taro, *Geosiphon* and *Archaeospora* for *Pterocarpus* adult trees. In addition, the significant differences observed between *Pterocarpus* adult trees and seedlings confirmed the modification of AM fungal communities according to the plant age (Wubet et al. 2009). Our data corroborate previous molecular studies conducted in tropical forests where divergent AM fungal communities of co-occurring plant species were reported (Uhlmann et al. 2004, Wubet et al. 2009, Mangan et al. 2010). However, three highly abundant AM fungal taxa (80% of sequences) were associated to the three plant types, notably *Funneliformis* (OTU1) that is considered as a generalist AM fungus (Öpik et al. 2006). *Funneliformis* was shown to form CMNs for transport of N, particularly in tropical environments where N is poorly available (Walder et al. 2012, Munroe and Isaac 2014). CMNs might play a major role in the sustainability of *Pterocarpus*-taro agroforestry systems and high crop yields. First, the CMNs maintained by *Pterocarpus* could provide an AM fungal mycelium reservoir enabling a faster colonization of short-lived crops under swamp forests (Kuyper et al. 2004) compared to an AM spore reservoir (Brundrett and Abbott 1994). Secondly, the CMNs could be involved in the N transfers from *Pterocarpus* trees or seedlings to taro in N-deficient soils of swamp forests, as observed between other legume and non-legume associations (Thilakarathna et al. 2016).

*Funneliformis* has been however described as a ruderal and stress tolerator taxa mainly involved in plant protection against biotic and abiotic stress rather than in plant nutrition (competitor) (Chagnon et al. 2013). Indeed, *Funneliformis* was shown to protect tropical plants against certain pathogens (Cardoso and Kuyper 2006). Several strategies could be set up to experimentally test the significance of *Funneliformis* in nutrient and/or plant protection in systems associating *Pterocarpus-*taro. Compartmented microcosms with inoculated *Funneliformis* strains and the use of a ^15^N-labeled growth substrate as designed in Walder et al. (2012) could be used to demonstrate (i) the N transfers through *Funneliformis* CMNs between the two studied plants, but also (ii) the benefits of *Pterocarpus-*taro associations compared to taro monoculture by measuring in parallel plant growth parameters. The introduction of a pathogen in the inoculated or not inoculated compartmented microcosms in parallel to the monitoring of plant N nutrition could be used to evaluate the significance of *Funneliformis* strains regarding (i) plant protection, but also (ii) the contribution of *Funneliformis* as competitor or ruderal (Chagnon et al. 2013). In the case of a decrease of *Funneliformis*-mediated plant N uptake, and the allocation of AM resources in plant protection, the status of *Funneliformis* as ruderal would be confirmed.

**Conclusions**

Our study highlights the high AM fungal diversity and richness associated with roots of *Pterocarpus* (adult trees and seedlings) and taro. Although the AM fungal community is significantly different in terms of membership and structure between the types of plants, *Pterocarpus* and taro had few but predominant overlapping AM fungi, notably *Funneliformis* spp. (OTU1). From an agricultural point of view, in addition to the good tolerance of taro to waterlogging and shade under *Pterocarpus* swamp forests (Saur and Imbert 2003), the preservation of *Pterocarpus* adult trees and their seedlings could be one of the main factors leading to high taro crop yields by maintaining N input on soil and a source of AM fungal inoculums that might form potential CMNs crucial for the establishment of taro.

**Acknowledgements**

We thank Fonds Européen de Développement Régional (FEDER) for their financial support and the Région Guadeloupe for support of Al.G.'s post-doctoral fellowship. We are grateful to A. Omrane and V. Virapin for their helpful contribution. We thank X. Salducci (LCA, Mauguio) for the soils analyses. The comments of two anonymous reviewers helped improve this manuscript.

**Author contributions**

A.B., An.G. designed the research and collected the samples. Al.G., H.S. developed the methodology and performed statistical analyses. Al.G., H.S. generated data. A.B., Al.G., and H.S. wrote the initial manuscript. A.B., Al.G., An.G. and H.S. contributed to the final manuscript. All the authors shared, edited and approved the final manuscript.

Conflict of Interest Statement: The authors declare that the research was conducted in the absence of any commercial or financial relationships that could be construed as a potential conflict of interest.

**Data**

Raw data are available under the BioProject ID PRJNA384862 (https://www.ncbi.nlm.nih.gov/bioproject).

**References**

Alguacil, M., Torres, P., Montesinos-navarro, A., and Rodan, A. (2016). Soil characteristics driving arbuscular mycorrhizal fungal communities in semiarid Mediterranean soils. Appl. Environ. Microbiol. 82, 3348–3356. doi:10.1128/AEM.03982-15.Editor.

Anderson, M. (2001). A new method for non parametric multivariate analysis of variance. Austral Ecol. 26, 32–46. Available at: http://onlinelibrary.wiley.com/doi/10.1111/j.1442-9993.2001.01070.pp.x/full.

Bâ, A. M., and Rivera-Ocasio, E. (2015). Genetic diversity and functional traits of *Pterocarpus officinalis* Jacq. associated with symbiotic microbial communities in Caribbean swamp forests in relation to insular distribution, salinity and flooding. *Wetlands* 35, 433–442. doi:10.1007/s13157-015-0651-5.

Bainard, L. D., Koch, A. M., Gordon, A. M., Newmaster, S. G., Thevathasan, N. V., and Klironomos, J. N. (2011). Influence of trees on the spatial structure of arbuscular mycorrhizal communities in a temperate tree-based intercropping system. *Agric. Ecosyst. Environ.* 144, 13–20. doi:10.1016/j.agee.2011.07.014.

Bálint, M., Bahram, M., Eren, A. M., Faust, K., Fuhrman, J. A., Lindahl, B., et al. (2016). Millions of reads, thousands of taxa: Microbial community structure and associations analyzed via marker genes. FEMS Microbiol. Rev. 40, 686–700. doi:10.1093/femsre/fuw017.

Boneh, S., Boneh, A., and Caron, R. (1998). Estimating the prediction function and the number of unseen species in sampling with replacement. *J. Am. Stat. Assoc.* 93, 372–379. doi:10.1080/01621459.1998.10474118.

Brearley, F. Q., Elliott, D. R., Iribar, A., and Sen, R. (2016). Arbuscular mycorrhizal community structure on co-existing tropical legume trees in French Guiana. *Plant Soil* 403, 253–265. doi:10.1007/s11104-016-2818-0.

Brundrett, M. C., and Abbott, L. K. (1994). Mycorrhizal fungus propagules in the Jarrah forest .1. Seasonal study of inoculum levels. *New Phytol.* 127, 539–546. doi:Doi 10.1111/J.1469-8137.1994.Tb03972.X.

Cardoso, I., and Kuyper, T. (2006). Mycorrhizas and tropical soil fertility. *Agric. Ecosyst. Environ.* 116, 72–84. doi:10.1016/j.agee.2006.03.011.

Chen, H. (2016). VennDiagram: Generate high-resolution venn and euler plots. R package version 1.6.17. https://CRAN.R-project.org/package=VennDiagram.

Cheng, X., and Baumgartner, K. (2004). Arbuscular mycorrhizal fungi-mediated nitrogen transfer from vineyard cover crops to grapevines. *Biol. Fertil. Soils* 40, 406–412. doi:10.1007/s00374-004-0797-4.

Davison, J., Moora, M., Öpik, M., Adholeya, A., Ainsaar, L., Bâ, A., et al. (2015). Global assessment of arbuscular mycorrhizal fungus diversity reveals very low endemism. Science. 349, 970–973. doi:10.5061/dryad.2m15n.

Davison, J., Öpik, M., Zobel, M., Vasar, M., Metsis, M., and Moora, M. (2012). Communities of arbuscular mycorrhizal fungi detected in forest soil are spatially heterogeneous but do not vary throughout the growing season. *PLoS One* 7. doi:10.1371/journal.pone.0041938.

De Beenhouwer, M., Van Geel, M., Ceulemans, T., Muleta, D., Lievens, B., and Honnay, O. (2015). Changing soil characteristics alter the arbuscular mycorrhizal fungi communities of Arabica coffee (*Coffea arabica*) in Ethiopia across a management intensity gradient. *Soil Biol. Biochem.* 91, 133–139. doi:10.1016/j.soilbio.2015.08.037.

De Cáceres, M., and Legendre, P. (2009). Associations between species and groups of sites: indices and statistical inference. Ecology 90, 3566–3574. doi:10.1890/08-1823.1.

De Cáceres, M., Legendre, P., and Moretti, M. (2010). Improving indicator species analysis by combining groups of sites. *Oikos* 119, 1674–1684. doi:10.1111/j.1600-0706.2010.18334.x.

De Cáceres, M., Legendre, P., Wiser, S. K., and Brotons, L. (2012). Using species combinations in indicator value analyses. *Methods Ecol. Evol.* 3, 973–982. doi:10.1111/j.2041-210X.2012.00246.x.

Dormann, C. F., Gruber, B., and Fründ, J. (2008). Introducing the bipartite package: Analysing ecological networks. R News 8, 8–11. doi:10.1159/000265935.

Edgar, R. C., Haas, B. J., Clemente, J. C., Quince, C., and Knight, R. (2011). UCHIME improves sensitivity and speed of chimera detection. *Bioinformatics* 27, 2194–2200. doi:10.1093/bioinformatics/btr381.

Eusse, A. M., and Aide, T. M. (1999). Patterns of litter production across a salinity gradient in a *Pterocarpus officinalis* tropical wetland. *Plant Ecol.* 145, 307–315.

Fougnies, L., Renciot, S., Muller, F., Plenchette, C., Prin, Y., de Faria, S. M., et al. (2007). Arbuscular mycorrhizal colonization and nodulation improve flooding tolerance in *Pterocarpus officinalis* Jacq. seedlings. *Mycorrhiza* 17, 159–166. doi:10.1007/s00572-006-0085-2.

Gihring, T. M., Green, S. J., and Schadt, C. W. (2012). Massively parallel rRNA gene sequencing exacerbates the potential for biased community diversity comparisons due to variable library sizes. *Environ. Microbiol.* 14, 285–290. doi:10.1111/j.1462-2920.2011.02550.x.

Hart, M. M., Aleklett, K., Chagnon, P., Egan, C., Ghignone, S., Helgason, T., et al. (2015). Navigating the labyrinth : a guide to sequence-based, community ecology of arbuscular mycorrhizal fungi. *New Phytol.* 207, 235–247. doi:10.1111/nph.13340.

He, X., Critchley, C., Ng, H., and Bledsoe, C. (2004). Nodulated N2-fixing *Casuarina cunninghamiana* is the sink for net N transfer from non-N2-fixing *Eucalyptus maculata* via an ectomycorrhizal fungus *Pisolithus* sp. using 15NH4+ or 15NO3− supplied as ammonium nitrate. *New Phytol.* 167, 897–912. doi:10.1111/j.1469-8137.2005.01437.x.

Helgason, T., and Fitter, A. H. (2009). Natural selection and the evolutionary ecology of the arbuscular mycorrhizal fungi (Phylum Glomeromycota). *J. Exp. Bot.* 60, 2465–2480. doi:10.1093/jxb/erp144.

Hervé, M. (2016). RVAideMemoire: Diverse basic statistical and graphical functions. R package version 0.9-60. <https://CRAN.R-project.org/package=RVAideMemoire>.

Hodge, A., Campbell, C. D., and Fitter, A. H. (2001). An arbuscular mycorrhizal fungus accelerates decomposition and acquires nitrogen directly from organic material. Nature 413, 297–299.

Holste, E. K., Holl, K. D., Zahawi, R. A., and Kobe, R. K. (2016). Reduced aboveground tree growth associated with higher arbuscular mycorrhizal fungal diversity in tropical forest restoration. *Ecol. Evol.* 6, 7253–7262. doi:10.1002/ece3.2487.

Huse, S. M., Welch, D. M., Morrison, H. G., and Sogin, M. L. (2010). Ironing out the wrinkles in the rare biosphere through improved OTU clustering. *Environ. Microbiol.* 12, 1889–1898. doi:10.1111/j.1462-2920.2010.02193.x.

Ingleby, K., Wilson, J., Munro, R. C., and Cavers, S. (2007). Mycorrhizas in agroforestry: Spread and sharing of arbuscular mycorrhizal fungi between trees and crops: complementary use of molecular and microscopic approaches. *Plant Soil* 294, 125–136. doi:10.1007/s11104-007-9239-z.

Kaiser, C., Kilburn, M. R., Clode, P. L., Fuchslueger, L., Koranda, M., Cliff, J. B., et al. (2015). Exploring the transfer of recent plant photosynthates to soil microbes: mycorrhizal pathway *vs* direct root exudation. *New Phytol.* 205, 1537–1551. doi:10.1111/nph.13138.

Koponen, P., Nygren, P., Domenach, A. M., Le Roux, C., Saur, E., and Roggy, J. C. (2003). Nodulation and dinitrogen fixation of legume trees in a tropical freshwater swamp forest in French Guiana. *J. Trop. Ecol.* 19, 655–666. doi:10.1017/s0266467403006059.

Krüger, M., Krüger, C., Walker, C., Stockinger, H., and Schüßler, A. (2012). Phylogenetic reference data for systematics and phylotaxonomy of arbuscular mycorrhizal fungi from phylum to species level. *New Phytol.* 193, 970–984. Available at: http://0-eds.a.ebscohost.com.serlib0.essex.ac.uk/eds/pdfviewer/pdfviewer?sid=2afd8315-eb95-4bfe-9f92-44b70f093aeb%40sessionmgr4010&vid=1&hid=4111.

Kuyper, T. W., Cardoso, I. M., Onguene, N. A., Van Noordwijk, M., and Van Noordwijk, M. (2004). “Managing below-ground interactions in agroecosystems,” in *Below-Ground Interactions in Tropical Agroecosystems* (Wallingford), 243–261.

Le Roux, C., Muller, F., Bouvet, J. M., Dreyfus, B., Béna, G., Galiana, A., et al. (2014). Genetic diversity patterns and functional traits of *Bradyrhizobium* strains associated with *Pterocarpus officinalis* Jacq. in caribbean islands and amazonian forest (French Guiana). *Microb. Ecol.* 68, 329–338. doi:10.1007/s00248-014-0392-7.

Leal, P. L., Siqueira, J. O., and Stürmer, S. L. (2013). Switch of tropical Amazon forest to pasture affects taxonomic composition but not species abundance and diversity of arbuscular mycorrhizal fungal community. *Appl. Soil Ecol.* 71, 72–80. doi:10.1016/j.apsoil.2013.05.010.

Lee, J., Lee, S., and Young, J. P. W. (2008). Improved PCR primers for the detection and identification of arbuscular mycorrhizal fungi. *FEMS Microbiol. Ecol.* 65, 339–349. doi:10.1111/j.1574-6941.2008.00531.x.

Mangan, S. a, Herre, E. a, and Bever, J. D. (2010). Specificity between Neotropical tree seedlings and their fungal mutualists leads to plant — soil feedback. *Ecology* 91, 2594–2603. doi:10.2307/27860837.

McArdle, B. H., and Anderson, M. J. (2001). Fitting multivariate models to community data: a comment on distance-based redundancy analysis. *Ecology* 82, 290–297. doi:10.1890/0012-9658(2001)082[0290:FMMTCD]2.0.CO;2.

Montesinos-Navarro, A., Segarra-Moragues, J. G., Valiente-Banuet, A., and Verdú, M. (2012). Plant facilitation occurs between species differing in their associated arbuscular mycorrhizal fungi. *New Phytol.* 196, 835–844. doi:10.1111/j.1469-8137.2012.04290.x.

Moora, M., Davison, J., Öpik, M., Metsis, M., Saks, Ü., Jairus, T., et al. (2014). Anthropogenic land use shapes the composition and phylogenetic structure of soil arbuscular mycorrhizal fungal communities. *FEMS Microbiol. Ecol.* 90, 609–621. doi:10.1111/1574-6941.12420.

Muller, F., Voccia, M., Bâ, A., and Bouvet, J. M. (2009). Genetic diversity and gene flow in a Caribbean tree *Pterocarpus officinalis* Jacq.: A study based on chloroplast and nuclear microsatellites. *Genetica* 135, 185–198. doi:10.1007/s10709-008-9268-4.

Munroe, J. W., and Isaac, M. E. (2014). N_2_-fixing trees and the transfer of fixed-N for sustainable agroforestry: A review. *Agron. Sustain. Dev.* 34, 417–427. doi:10.1007/s13593-013-0190-5.

Oehl, F., Laczko, E., Bogenrieder, A., Stahr, K., Bösch, R., van der Heijden, M., et al. (2010). Soil type and land use intensity determine the composition of arbuscular mycorrhizal fungal communities. *Soil Biol. Biochem.* 42, 724–738. doi:10.1016/j.soilbio.2010.01.006.

Oksanen, J., Blanchet, F.G., Friendly, M., Kindt, R., Legendre, P., McGlinn, D., Minchin, P.R., O'Hara, R. B., Simpson, G.L., Solymos, P., Stevens, M.H.H., Szoecs, E., and Wagner, H. (2016). vegan: community ecology package. R package version 2.4-1. https://CRAN.R-project.org/package=vegan.

Öpik, M., Davison, J., Moora, M., and Zobel, M. (2014). DNA-based detection and identification of Glomeromycota : the virtual taxonomy of environmental sequences. *Botany* 92, 135–147. doi:10.1139/cjb-2013-0110.

Öpik, M., Moora, M., Liira, J., and Zobel, M. (2006). Composition of root-colonizing arbuscular mycorrhizal fungal communities in different ecosystems around the globe. *J. Ecol.* 94, 778–790. doi:10.1111/j.1365-2745.2006.01136.x.R Core Team (2016). R: A language and environment for statistical computing. R Foundation for Statistical Computing, Vienna, Austria. https://www.R-project.org/.

Rivera-Ocasio, E., Aide, T. M., and McMillan, W. O. (2006). The influence of spatial scale on the genetic structure of a widespread tropical wetland tree, *Pterocarpus officinalis* (Fabaceae). *Conserv. Genet.* 7, 251–266. doi:10.1007/s10592-005-9022-8.

Rodríguez-Echeverría, S., Teixeira, H., Correia, M., Timóteo, S., Heleno, R., Öpik, M., et al. (2016). Arbuscular mycorrhizal fungi communities from tropical Africa reveal a strong spatial structure. *Cardoso, I., and Kuyper, T. (2006). Mycorrhizas and tropical soil fertility. Agric. Ecosyst. Environ. 116, 72–84. doi:10.1016/j.agee.2006.03.011.*

Saint-Etienne, L., Paul, S., Imbert, D., Dulormne, M., Muller, F., Toribio, A., et al. (2006). Arbuscular mycorrhizal soil infectivity in a stand of the wetland tree *Pterocarpus officinalis* along a salinity gradient. *For. Ecol. Manage.* 232, 86–89. doi:10.1016/j.foreco.2006.05.046.

Sanguin, H., Mathaux, C., Guibal, F., Prin, Y., Mandin, J. P., Gauquelin, T., et al. (2016). Ecology of vertical life in harsh environments: The case of mycorrhizal symbiosis with secular cliff climbing trees (*Juniperus phoenicea* L.). *J. Arid Environ.* 134, 132–140. doi:10.1016/j.jaridenv.2016.07.008.

Sanou, J., Bayala, J., Teklehaimanot, Z., and Bazié, P. (2012). Effect of shading by baobab (*Adansonia digitata*) and néré (*Parkia biglobosa*) on yields of millet (*Pennisetum glaucum*) and taro (*Colocasia esculenta*) in parkland systems in Burkina Faso, West Africa. *Agrofor. Syst.* 85, 431–441. doi:10.1007/s10457-011-9405-4.

Saur, E., and Imbert, D. (2003). Traditional taro monoculture in the swamp forest of Guadeloupe. *Bois Forets des Trop.* 277, 85–89.

Schloss, P. D., Gevers, D., and Westcott, S. L. (2011). Reducing the effects of PCR amplification and sequencing artifacts on 16s rRNA-based studies. *PLoS One* 6, e27310. doi:10.1371/journal.pone.0027310.

Selosse, M. A., Richard, F., He, X., and Simard, S. W. (2006). Mycorrhizal networks: des liaisons dangereuses? *Trends Ecol. Evol.* 21, 621–628. doi:10.1016/j.tree.2006.07.003.

Simon, L., Lalonde, M., and Bruns, T. (1992). Specific amplification of 18S fungal ribosomal genes from vesicular-arbuscular endomycorrhizal fungi colonizing roots. *Appl. Environ. Microbiol.* 58, 291–295.

Smith, S. E., and Read, D. J. (2008). *Mycorrhizal symbiosis*. Academic Press.

Suz, L. M., Barsoum, N., Benham, S., Dietrich, H. P., Fetzer, K. D., Fischer, R., et al. (2014). Environmental drivers of ectomycorrhizal communities in Europe’s temperate oak forests. *Mol. Ecol.* 23, 5628–5644. doi:10.1111/mec.12947.

Thilakarathna, M. S., McElroy, M. S., Chapagain, T., Papadopoulos, Y. A., and Raizada, M. N. (2016). Belowground nitrogen transfer from legumes to non-legumes under managed herbaceous cropping systems. A review. *Agron. Sustain. Dev.* 36, 1–16. doi:10.1007/s13593-016-0396-4.

Thioye, B., Mania, S., Kane, A., Ndiaye, C., Ould Soule, A., Fall, D., et al. (2017). Growth response of different species of *Ziziphus* to inoculation with arbuscular mycorrhizal fungi. Fruits 72, 174–181. doi:10.17660/th2017/72.3.7.

Uhlmann, E., Görke, C., Petersen, A., and Oberwinkler, F. (2004). Arbuscular mycorrhizae from semiarid regions of Namibia. *Can. J. Bot.* 82, 645–653. doi:10.1016/j.jaridenv.2005.05.002.

Varela-Cervero, S., Vasar, M., Davison, J., Barea, J. M., Öpik, M., and Azcón-Aguilar, C. (2015). The composition of arbuscular mycorrhizal fungal communities differs among the roots, spores and extraradical mycelia associated with five Mediterranean plant species. *Environ. Microbiol.* 17, 2882–2895. doi:10.1111/1462-2920.12810.

Wahbi, S., Maghraoui, T., Hafidi, M., Sanguin, H., Oufdou, K., Prin, Y., et al. (2016). Enhanced transfer of biologically fixed N from faba bean to intercropped wheat through mycorrhizal symbiosis. *Appl. Soil Ecol.* 107, 91–98. doi:10.1016/j.apsoil.2016.05.008.

Walder, F., Niemann, H., Natarajan, M., Lehmann, M. F., Boller, T., and Wiemken, A. (2012). Mycorrhizal networks: common goods of plants shared under unequal terms of trade. *Plant Physiol.* 159, 789–797. doi:10.1104/pp.112.195727.

Wang, B., and Qiu, Y. L. (2006). Phylogenetic distribution and evolution of mycorrhizas in land plants. *Mycorrhiza* 16, 299–363. doi:10.1007/s00572-005-0033-6.

Wubet, T., Kottke, I., Teketay, D., and Oberwinkler, F. (2009). Arbuscular mycorrhizal fungal community structures differ between co-occurring tree species of dry Afromontane tropical forest, and their seedlings exhibit potential to trap isolates suited for reforestation. *Mycol. Prog.* 8, 317–328. doi:10.1007/s11557-009-0602-8.

Wubet, T., Weiß, M., Kottke, I., Teketay, D., and Oberwinkler, F. (2003). Molecular diversity of arbuscular mycorrhizal fungi in *Prunus africana*, an endangered medicinal tree species in dry Afromontane forests of Ethiopia. *New Phytol.* 161, 517–528. doi:10.1046/j.1469-8137.2003.00924.x.

**Legends**

**Figure 1**. Abundance of AM fungal OTUs between Grande Ravine and Belle Plaine forest sites. Only OTUs with significant (*P* < 0.05) differences in their relative abundance between the both forest sites are shown. Table represented comparison of AM fungal OTU abundances between localities (Belle Plaine and Grande Ravine) with statistics were performed using Kruskal-Wallis' test. ‘*’ P < 0.05.

**Figure 2**. Distribution of AM fungal OTUs shared among taro (T), *Pterocarpus* adult trees (P.a) and seedlings (P.s) in Grande Ravine and Belle Plaine forest sites. All sequences were clustered in OTUs (97% similarity). Data were rarefied to 440 sequences per sample. (A) Venn diagrams represent the percentage of AM fungal OTUs specific to a given plant or overlapping among plants. (B) Bipartite network represents the abundance of each OTU and the number and level of connections to each plant.AM fungi. Each box in the bottom level represents an AM fungal OTU which is linked to plants (boxes on the top level). The width of each AM fungal box is proportional to the number of reads for each OTU. The width of each connection is proportional to the number of reads connected to the plant. AM fungal OTUs are sorted from left to right by ascending order (see Table S3 for OTUs taxonomy).

**Figure S1**. Map of Guadeloupe with the two sampling forest sites.

**Figure S2**. Nonmetric multidimensional scaling (NMDS) ordination of *Pterocarpus* and taro root-associated AM fungal communities in Grande Ravine and Belle Plaine forest sites. Different colors represented the three types of plants.

**Table 1.**

Impact of locality and plant type on AM fungal community structures in agroforestry systems.

**Table 2**. Single of

AM fungal OTUs associated with a plant type in *Pterocarpus*-taro in Guadeloupean agroforestry systems.

**Table S1**. Soil characteristics of forest sites

**Table S2**. Richness and diversity of AM fungal communities associated with taro and *Pterocarpus* in agroforestry systems.

**Table S3**. Taxonomy assignment of 215 OTUs identified in *Pterocarpus*-taro based agroforestry systems.

^1^ The number between () indicate the confidence threshold for the OTU assignment.

**Table S4**. Comparison of AM fungal OTU abundances between localities (Grande Ravine and Belle Plaine).

^2^ statistics were performed using Kruskal-Wallis' test. ‘*’ P < 0.05; ‘ns’ P > 0.05. In bold OTUs presenting a significant difference between the two sites.

**Table S5.** AM fungal OTUs preferentially associated with a locality (Grande Ravine or Belle Plaine)

**Table S6**. Single and pair of AM fungal OTUs associated with a plant type in *Pterocarpus*-taro in Guadeloupean agroforestry systems.


**Table 1.** Impact of locality and plant type on AM fungal community structures in agroforestry systems.

| Locality | Factors | Df | SS | MS | F. Model | R^2^ / N perm | P-value ^1^ |
| --- | --- | --- | --- | --- | --- | --- | --- |
| All | Locality | 1 | 0.11671 | 0.11671 | 1.298 | 0.05730 | 0.242 ^ns^ |
|  | Plant | 2 | 0.26401 | 0.132006 | 1.4610 | 0.12962 | 0.173 ^ns^ |
|  | Locality × Plant | 2 | 0.57188 | 0.285939 | 3.1647 | 0.28077 | **0.011*** |
|  | Residuals | 12 | 1.08422 | 0.090352 |  | 0.53231 |  |
|  | Total | 17 | 2.03682 |  |  | 1 |  |
|  |  |  |  |  |  |  |  |
| Belle Plaine | Plant | 2 | 0.38777 | 0.19389 | 1.4766 | 0.32984 | 0.079 ^ns^ |
|  | Residuals | 6 | 0.78786 | 0.13131 |  | 0.670.16 |  |
|  | Total | 8 | 1.17563 |  |  | 1.00000 |  |
|  |  |  |  |  |  |  |  |
| Grande Ravine | Plant | 2 | 0.44812 | 0.224058 | 4.5362 | 0.60192 | **0.036 ^*^** |
|  | Residuals | 6 | 0.29636 | 0.049393 |  | 0.39808 |  |
|  | Total | 8 | 0.74448 |  |  | 1.00000 |  |

^1^ The significance of multivariate analysis of variance was assessed using PERMANOVA with *adonis*() function (iterations = 999 permutations). ‘*’ *P* < 0.05; ‘ns’ *P* > 0.05. Multivariate dispersion was tested using the *betadisper*() and *permutest*() functions (iterations = 999 permutations; alpha = 0.05) revealing a significant homogeneity of group dispersions.


**Table 2**. Single of AM fungal OTUs associated with a plant type in *Pterocarpus*-taro in Guadeloupean agroforestry systems.

| *Locality* | *Plant* | *Indicator taxa* | *Frequency ^1^* | *A ^1^* | *B* | *IndVal.g* | *P-value ^3^* |
| --- | --- | --- | --- | --- | --- | --- | --- |
|  |  | *Taxonomy (OTU)* | *(P.a / P.s / T)* | *(specificity)* | *(sensibility)* |  |  |
| *Belle Plaine* | *T.* | *Gigaspora (33)* | *0.0 / 0.0 / 1.0* | *1.00* | *1.00* | *1.00* | *0.027^*^* |


| *Grande Ravine* | *T.* | *Gigaspora (22)* | *0.0 / 0.0 / 1.0* | *1.00* | *1.00* | *1.00* | *0.035^*^* |
| --- | --- | --- | --- | --- | --- | --- | --- |


|  | *P.a* | *Geosiphon (18)* | *0.0 / 0.0 / 1.0* | *1.00* | *1.00* | *1.00* | *0.032^*^* |
| --- | --- | --- | --- | --- | --- | --- | --- |
|  |  | *Archaeospora (10)* | *1.0 / 0.0 / 0.3* | *0.96* | *1.00* | *0.98* | *0.032^*^* |
|  | *T. + P.s* | *Incertae sedis Glomus (4)* | *0.0 / 1.0 / 1.0* | *1.00* | *1.00* | *1.00* | *0.032^*^* |


|  |  | *Rhizophagus (23)* | *0.0 / 1.0 / 1.0* | *1.00* | *1.00* | *1.00* | *0.032^*^* |
| --- | --- | --- | --- | --- | --- | --- | --- |


^1^ Frequency indicates the presence of an OTU in samples of a given plant type. P.a, *Pterocarpus* adult tree; P.s, *Pterocarpus* seedling; T., taro

^3^ The corrected Indval coefficient of association (“IndVal.g”) was used as model to determine indicator OTUs.

^3^ ‘*’ *P* < 0.05; ‘ns’ *P* > 0.05
